# Surfactant-loaded capsules as Marangoni microswimmers at the air–water interface: Symmetry breaking and spontaneous propulsion by surfactant diffusion and advection

**DOI:** 10.1140/epje/s10189-021-00035-8

**Published:** 2021-03-08

**Authors:** Hendrik Ender, Ann-Kathrin Froin, Heinz Rehage, Jan Kierfeld

**Affiliations:** 1 Department of Physics, Technische Universität Dortmund, 44221 Dortmund, Germany; 2 Department of Chemistry and Chemical Biology, Technische Universität Dortmund, 44221 Dortmund, Germany

## Abstract

**Abstract:**

We present a realization of a fast interfacial Marangoni microswimmer by a half-spherical alginate capsule at the air–water interface, which diffusively releases water-soluble spreading molecules (weak surfactants such as polyethylene glycol (PEG)), which act as “fuel” by modulating the air–water interfacial tension. For a number of different fuels, we can observe symmetry breaking and spontaneous propulsion although the alginate particle and emission are isotropic. The propulsion mechanism is similar to soap or camphor boats, which are, however, typically asymmetric in shape or emission to select a swimming direction. We develop a theory of Marangoni boat propulsion starting from low Reynolds numbers by analyzing the coupled problems of surfactant diffusion and advection and fluid flow, which includes surfactant-induced fluid Marangoni flow, and surfactant adsorption at the air–water interface; we also include a possible evaporation of surfactant. The swimming velocity is determined by the balance of drag and Marangoni forces. We show that spontaneous symmetry breaking resulting in propulsion is possible above a critical dimensionless surfactant emission rate (Peclet number). We derive the relation between Peclet number and swimming speed and generalize to higher Reynolds numbers utilizing the concept of the Nusselt number. The theory explains the observed swimming speeds for PEG–alginate capsules, and we unravel the differences to other Marangoni boat systems based on camphor, which are mainly caused by surfactant evaporation from the liquid–air interface. The capsule Marangoni microswimmers also exhibit surfactant-mediated repulsive interactions with walls, which can be qualitatively explained by surfactant accumulation at the wall.

**Graphic Abstract:**

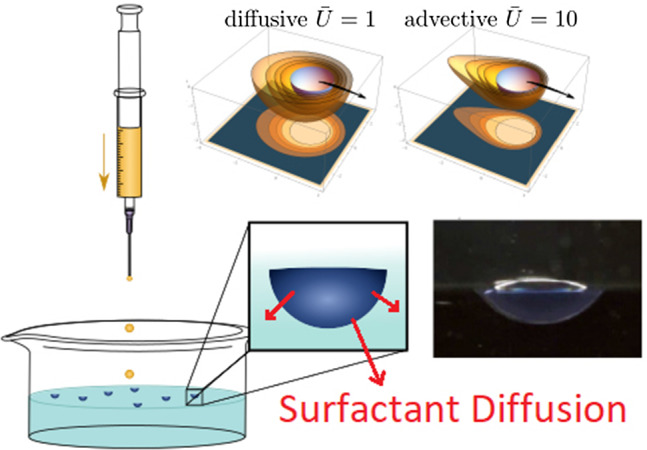

## Introduction

Designing and understanding self-propelling biological or artificial microswimmers is the basis for the physics of active systems. Swimming on the microscale is governed by low Reynolds numbers and requires special propulsion mechanisms which are effective in the presence of dominating viscous forces. The first propulsion principle that comes to mind is shape-changing swimmers, which deform their body in a cyclic way in order to propel. At low Reynolds numbers, the cyclic deformation pattern of a swimmer must not be invariant under time-reversal according to the scallop theorem [1]. In nature, many different examples of deformation swimmers can be found such as bacteria, algae and spermatozoa [2]. Realizing this concept in synthetic microswimmers is often difficult as the scallop theorem requires control of at least two parameters.

Shape-changing swimmers force the surrounding fluid via no-slip boundary conditions on the surface of their moving parts. Another successful class of synthetic microswimmers is phoretic swimmers, which actively create slip velocities at their surface. Self-propelling phoretic swimmers autonomously create gradients in external fields such as concentration of a “fuel” or temperature, which in turn give rise to symmetry-breaking interfacial fluid flow in a thin interaction layer [3]. This fluid flow constitutes an effective slip velocity leading to propulsion [4, 5]. Examples of such autophoretic swimmers are thermophoretic or diffusiophoretic swimmers, which generate gradients in temperature or concentration of interacting particles along their body. Self-diffusiophoretic swimmers generate a non-vanishing interfacial slip velocity on the particle surface via asymmetries in the solute concentration field and a short-range interaction between solute and swimmer [3]. Diffusiophoretic models typically neglect advection of the fuel concentration [6–8], but this has been included in Refs. [9, 10]. A lot of different aspects of swimmer behavior have been studied for self-diffusiophoretic swimmers such as efficiency [11], confinement effects [6, 8] cargo transport [7, 12] or the rich behavior during collisions with walls [13, 14].

While diffusiophoresis creates concentration gradients within the liquid surrounding the swimmer, concentration gradients or surface active molecules (surfactants) within the interface of *liquid* swimmers can also generate symmetry-breaking interfacial forces based on the Marangoni effect [15]. These propulsion mechanism based on the Marangoni effects are utilized in different liquid Marangoni swimmers, such as active liquid droplets or active emulsions [16]. Examples are pure water in an oil–surfactant medium (squalane and monoolein) [17] or liquid crystal droplets in surfactant solutions [16] but many other systems can be generated making this a versatile route to microswimmer production. This type of Marangoni swimmer is a liquid drop fully immersed in a liquid carrying surfactant, and propulsion is generated by the Marangoni effect along the liquid–liquid interface between swimmer and surrounding liquid, where a surfactant concentration gradient is maintained. In Ref. [17], an auto-diffusiophoretic mechanism [9, 18] has been proposed to maintain the surfactant concentration gradient. Another mechanism that has been proposed is increased adsorption of surfactant at the front (in swimming direction) of the swimmer, which depresses the interfacial tension in the front [16, 19, 20]. This gives rise to a Marangoni stress toward the back (where the interfacial tension is higher). The Marangoni stress forces the surrounding fluid toward the back of the swimmer resulting in a swimmer motion toward the front of the swimmer. For all proposed mechanisms, the liquid swimmer autonomously maintains an increased surfactant concentration in the front of its interface with the surrounding liquid, and it propels in the direction of *higher* surfactant concentration at its own interface.

The self-phoretic and Marangoni swimming mechanisms discussed so far do not generate net forces on the swimmer but non-vanishing slip velocities on the particle surface via asymmetries in a temperature or solute concentration field. There is another class of self-propelling swimmers partly based on the Marangoni effect and with a long history [21], which are so-called soap or camphor boats (or surfers), which we call *Marangoni boats* in the following. Marangoni boats are moving at the liquid–air interface [22]; typically, they are *solid* swimmers and operate at the centimeter scale. They are often used as a popular demonstration experiment for the Marangoni effect [23]. As “fuel” serve surface active molecules, which are deposited on the floating swimmer [23] or in which the swimmer is soaked [24–29], or the swimmer body itself is made from dissolving surfactant [30]. There are many examples based on DMF (dimethylformamide) [31], alcohol [23, 29], soap [29], camphor [24–28, 32] or camphene [30] that have also been characterized quantitatively. The surfactant molecules are emitted or dissolved from the swimmer and a radial concentration gradient is established at the air–water interface by diffusion, eventually aided by evaporation for volatile surfactants. The radial concentration gradient creates (i) surface tension gradients and (ii) Marangoni stresses on the fluid. This leads, however, not necessarily to swimming as long as the surface tension is symmetric and uniform around the swimmer. The surface tension is pulling in normal direction on the closed air–water–swimmer contact line. A uniform surface tension cancels along any arbitrarily shaped closed three-phase contact line, but a gradient in surfactant concentration along the contact line can generate a net propulsion force. We call this net force generated by surface tension gradients *direct Marangoni force* in the following. Also symmetry-broken Marangoni flows created by the Marangoni effect can contribute to (or impede) the propulsion via hydrodynamic drag onto the swimmer surface. We denote the resulting forces that Marangoni flows exert by *Marangoni flow forces* in the following. The Marangoni boat mechanism is relying on both types of forces. If surfactant emission is anisotropic the boat is, in general, propelled into the direction of higher surface tension, i.e., *lower* surfactant concentration along the air–water–swimmer contact line. We note that this is opposite to the propulsion in the direction of higher surfactant concentration for the active liquid swimmers discussed before. The Marangoni boat mechanism is also employed by some insects (rove beetle and *Velia*) [33] to propel on the water surface. There are also recent experiments [34] and theoretical work [35] on a closely related system of thermally driven Marangoni boats or surfers.

A full quantitative theory of Marangoni boats including hydrodynamics, surfactant advection, direct Marangoni forces and Marangoni flows is still elusive despite previous progress [22, 26, 36, 37]. Some theoretical approaches ignore the advection [35, 38, 39], several ignore the hydrodynamic flow fields [24, 25, 32, 40–42] or approximate it by uniform flow [28], which clearly oversimplifies the description of surfactant transport. In particular, on the numerical side, a recent paper of Kang et al. [37] provides progress by including advection fully into the numerical solution for an anisotropic Marangoni boat. A theoretical description is complicated by the fact that most of the Marangoni boats operate at higher Reynolds numbers, and fluid flow generated during Marangoni propulsion is typically featuring vortices [29, 37]. Miniaturization to the microscale leads to low Reynolds numbers. Therefore, miniaturization is not only attractive for possible applications but also provides a starting point for the development of hydrodynamic theories, as the simpler linear Stokes equation holds for fluid flow at low Reynolds numbers. This has been initiated in Refs. [35, 36, 38, 39].

Another question is regarding the role of intrinsic anisotropy, namely, whether isotropic swimmers with no intrinsically defined motion direction are also capable of a spontaneous motion which then spontaneously breaks the symmetry of the system. This question has been answered positively for autophoretic swimmers [9, 18], where it has been shown that advection by the surrounding fluid can maintain the necessary gradients in fields and/or concentrations above a critical strength of the advection (characterized by a dimensionless Peclet number). Liquid Marangoni swimmers are always symmetric by construction and have to maintain an increased surfactant concentration in the front of their interface by adsorption of surfactant (or micelles) or by autophoretic effects [17, 19, 20]. For the Marangoni boats the question regarding spontaneous symmetry breaking has been addressed experimentally in Ref. [28], where symmetric camphor disks have been shown to propel and swimming velocities have been shown to be largely independent of intrinsic swimmer anisotropy. So far, a theoretical answer is missing for Marangoni boats.

**Fig. 1:**
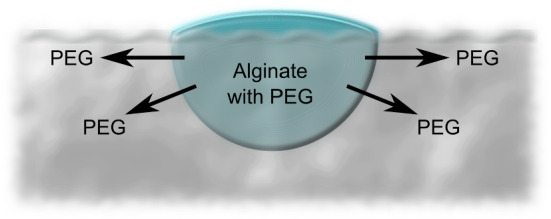
Schematic of the PEG–alginate capsule. The water-soluble “fuel” or spreading molecule PEG is incorporated during alginate capsule synthesis in the core and diffusively emitted during swimming

Here, we present a combined experimental and theoretical approach. We try to further approach the microscale by synthesizing alginate capsules as swimmer bodies, which provide a porous matrix that can accept surface active molecules. Several weakly surface active fuels are tested, among which polyethylene glycol (PEG) turns out to be the most effective. The PEG–alginate swimmers exhibit fast and prolonged propulsion. In general, we find prolonged propulsion only if spreading molecules are water-soluble as for PEG; then the air–water interface can regenerate by the fuel being dissolved in water. The PEG swimmers are approximately half-spherical, i.e., symmetric; therefore, we can address the question of spontaneous motion for a symmetric swimmer design. Moreover, a half-spherical shape turns out to be very convenient for theoretical modeling, and has also been employed in Ref. [37]. For small Reynolds numbers, this geometry allows for a complete theoretical description of Marangoni boat propulsion by analyzing the coupled problems of surfactant diffusion and advection, fluid flow, which includes surfactant-induced fluid Marangoni flow, and surfactant adsorption at the air–water interface; we also include a possible evaporation of surfactant. The swimming speed is determined from the balance of Marangoni forces (both direct forces from surface tension gradients and from Marangoni flow forces) and drag forces. We can address the problem of spontaneous symmetry breaking and predict the swimmer’s speed in a stationary state. This solution gives also hints how to generalize to higher Reynolds numbers using the concept of the Nusselt number, for which many results are known phenomenologically.

On the experimental side, we find further effects, such as the repulsive interaction of PEG–alginate swimmers with walls and the tendency to move in curved trajectories, which can be explained in the framework of the Marangoni boat mechanism.

## Alginate-based capsule swimmers

### Swimmer synthesis and characterization

The synthesized capsules show typical propelling mechanisms similar to phenomena observed for the insect class of *Microvelia*. Our artificial microswimmers consist of PEG droplets, which were surrounded by thin alginate shells (Fig. 1). For the preparation of these particles, we first form an aqueous PEG–alginate composite solution (standard: $w_\mathrm{PEG 300}=0.5\%$, $w_\mathrm{alginate}=0.5\%$). A droplet of this mixture is then deposed on the surface of an aqueous $\hbox {CaCl}_{2}$ solution (standard: $w_{\mathrm{CaCl}_{2}\cdot {2{\mathrm{H}}_{2}\mathrm{O}}}=0.5\%$). The ${\hbox {Ca}_{2+}}$ ions serve as cross-linker and induce, within several microseconds, the gelation of the alginate membranes according to the box-egg model [43–46]. Immediately after the formation of these particles, the capsules start to swim along the water surface.

**Fig. 2:**
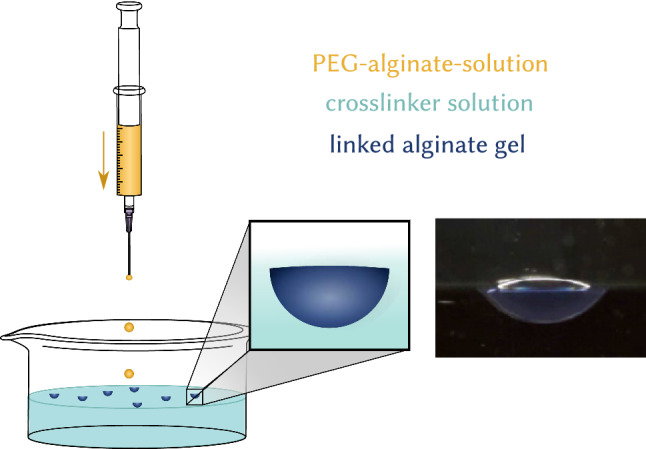
Synthesis of PEG–alginate swimmers by pipetting microliter amounts of PEG–alginate solution into cross-linker solution. Side view of a PEG–alginate swimmer showing its half-spherical shape

Dripping microliter amounts of alginate into a cross-linker salt solution containing counterions starts an ionotropic gelation and produces approximately half-spherical alginate gel capsules of millimeter radius (see Fig. 2) [43]. We report results for $a\sim 1500\,{\upmu \mathrm{m}}$; radii $a\sim 150\,{\upmu \mathrm{m}}$ can be reached. For alginate gelation, different salt solutions can be used containing divalent cations such as $\hbox {CaCl}_{2}$, $\hbox {CuCl}_{2}$, or $\hbox {BaCl}_{2}$ solutions.

**Table 1:** Fuel substances leading to successful alginate capsule propulsion

Polymers	Alcohols	Acids	Organic solvents
PEG 200	Ethylene glycol	Acetic acid	Acetone
PEG 300	Propylene glycol		Dimethyl sulfoxide
PEG 400	Diethylene glycol		Tetrahydrofuran
PEG 600	Ethanol		
PEG 1000	Isopropanol		
PEG 6000	1-pentanol		
PEG 20000	Benzyl alcohol		
PEG 35000	1-hexanol		
PPG 400	2-butanol, Tert-butanol		
	Dodecanol		

Adding surfactant to the alginate solution before dripping automatically loads the porous gel capsule with surfactant molecules. For suitable surfactants, capsules start to propel spontaneously on the air–water interface directly after dripping. The simple dripping technique allowed us to test many different “fuels”: successfully propelling fuels are polyethylene glycols (PEGs) with molar weights 200–35000 $\mathrm{g/mol}$, alcohols, acetic acid (stronger acids lead to protonization of alginate and subsequent coagulation), and organic solvents. A complete list of successfully tested fuels substances is given in Table 1. Swimmers fueled by PEG (or polypropylene glycol (PPG)), in particular PEG 300, exhibit the best results regarding propulsion speed and propulsion duration; the reason is a suitable combination of diffusion constant, solubility, but also gelation properties of the alginate–PEG mixture. Corresponding monomers and dimers (ethylene glycol, propylene glycol, diethylene glycol) also exhibit good swimming properties but with lower speed and duration. It is particularly important for a prolonged propulsion that the fuel substance lowers the air–water surface tension but is also water-soluble such that it dissolves in the water reservoir after spreading in order to regenerate the air–water interface. Evaporation from the air–water interface is another mechanism to achieve such a regeneration, which is at work in camphor boats [26–28, 32]. Strong surfactants and detergents, such as sodium dodecyl sulfate, generate spreading pressures that can rupture the alginate capsule. Moreover, they quickly saturate the air–water interface such that concentration gradients and, thus, swimming cannot be established. In the following, we report results for solutions of alginate and PEG 300 ($w_\mathrm{alginate}=0.5\%$ and $w_\mathrm{PEG 300}=0.5\%$) dripped into a $\hbox {CaCl}_{2}$ cross-linker solution ($w_{{\mathrm{CaCl}_{2}}\cdot {2\mathrm{H}_{2}{\mathrm{O}}}}=0.5\%$).

**Fig. 3:**
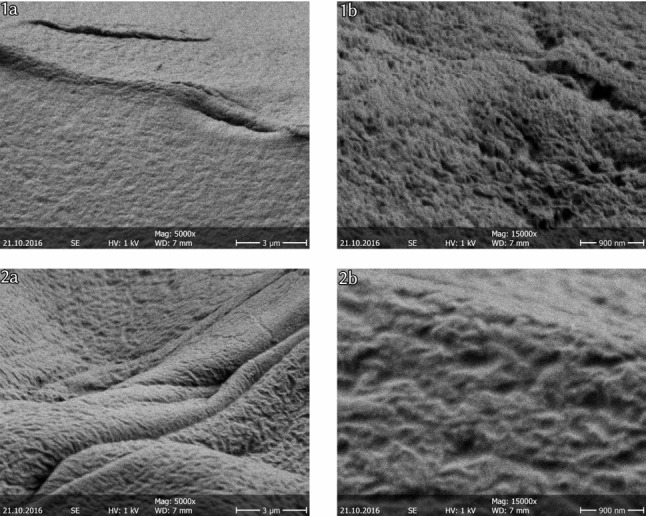
Scanning electron microscopy images of the porous structure of unloaded alginate capsules (1a,1b) and PEG-loaded alginate capsules (2a,2b) in 5000-fold (1a,2a) and 15000-fold (1b,2b) magnification

Alginate gels have a porous structure [43–45]. Scanning electron microscopy (SEM) of the alginate capsules reveals their porosity and also a certain roughness on the microscale with asperities on the capsule surface (see Fig. 3). The pores are essential for the slow diffusive emission of surfactant from the capsule [44, 45]. PEG diffusion through the porous alginate matrix is much slower than PEG diffusion in water; therefore, PEG should be released with a slowly varying controlled diffusive current that is limited by its slow diffusion in the alginate. The shape of the capsule and the spatial distribution of pores on the surface can break the overall spherical symmetry and give rise to small anisotropies in the emission, in principle.

### Swimming motion

**Fig. 4:**
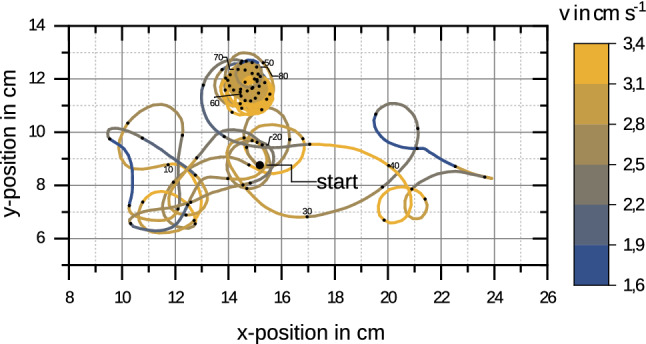
Typical swimming trajectory of a PEG–alginate swimmer in the cross-linker solution. Color-coded is the swimming velocity $U_\mathrm{swim}$, the trajectory shows the first $84\,\mathrm{s}$ of swimming

The alginate–PEG swimmers exhibit a fast and sustained motion. The swimming motion was observed in a cylindrical dish (diameter $24\,\mathrm{cm}$) for up to $20\,\mathrm{min}$. The swimmers exhibit typical speeds $U_\mathrm{swim} \sim 2-3\, \mathrm{cm/s}$ corresponding to 10–20 swimmer sizes per second (see Fig. 4); after $20\,\mathrm{min}$, velocities $U_\mathrm{swim} \sim 1 \,\mathrm{cm/s}$ can still be measured. This swimming performance is comparable to camphor boats [27, 28] and active liquid droplets [16, 17].

A typical swimming trajectory (lasting $84 \,\mathrm{s}$) far from a wall is shown in Fig. 4. We obtained this trajectory from a single-particle tracking analysis (using ImageJ); typical swimming velocities are $U_\mathrm{swim} \sim 2-3 \,\mathrm{cm/s}$ corresponding to $10-20$ swimmer sizes per second. This corresponds to moderate Reynolds numbers $\mathrm{Re} = {\rho U_\mathrm{swim} 2a}/{\mu }\sim 60$ (with the swimmer diameter $2a\simeq 3000\,{\upmu \mathrm{m}}$ as length scale and the viscosity and density of water, $\mu \simeq 10^{-3} \,\mathrm{Pa s}$ and $\rho = 10^3 \,\mathrm{kg/m^3}$).

**Fig. 5:**
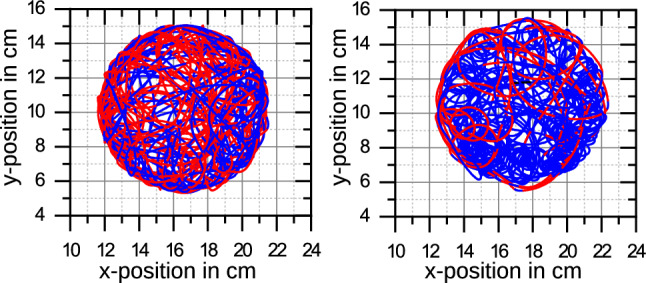
Swimming trajectory of two PEG–alginate swimmers in a cylindrical container. Color-coded is the sign of the curvature, blue/red trajectories curve clockwise/counter-clockwise. Swimmers prepared according to same protocol exhibit individually different curving behaviors (left: mostly counter-clockwise, right: mostly clockwise). Reflections at walls are of different duration

Swimming trajectories such as in Fig. 4 and in confinement in Fig. 5 exhibit phases with a characteristic curvature. Marking the swimmer with elongated plastic fragments shows that the elongated fragment is not turning with respect to the direction of motion, i.e., the curving of the trajectory is correlated with a reorientation of the swimmer. This is a hint that during curved swimming the swimming direction is linked to the orientation of the particle and, therefore, that the spherical symmetry is slightly broken by irregularities in the porous structure of the alginate particle (see SEM pictures in Fig. 3). The swimming direction is selected by dominating pores which determine a preferred direction of emission and, thus, propulsion by the resulting surfactant gradients. Curving itself can be caused by additional torques from asperities of the alginate capsule where surfactant is emitted preferentially in the tangential direction. A similar mechanism is at work at camphor-driven rotors [42, 47]. This is supported by the finding that the curving behavior of swimmers prepared by the same protocol (such as the swimmers in Fig. 5) is individually different and seems to depend on small differences between irregularities acquired in the preparation process. Recently, also vortex shedding at Reynolds numbers $\mathrm{Re}\sim$ 100–200 have been proposed to cause curving of trajectories [29].

Swimmers are also repelled by walls and reverse their direction of motion normal to the wall. In a course of a collision in normal direction, the swimmer keeps, however, its orientation while the direction of motion is reversed, i.e., during normal wall collisions the swimming direction also reverses with respect to the particle orientation. Swimming direction reversal has also been observed for camphor boats [22, 24, 25]. Swimming trajectories in Fig. 5 also show collisions with walls that last longer; these collisions can also feature a reorientation of swimmer, similar to what has been predicted for self-diffusiophoretic swimmers [13].

### Swimming mechanism

The order of magnitude of swimming speeds can only be explained as a result of a modulation of the large liquid–air surface tension. Marangoni mechanisms based on surface tension variations within the gel–liquid interface between alginate capsule and surrounding water are unlikely because the interfacial tensions and, thus, also Marangoni stresses, are too small for solid–liquid or gel–liquid interfaces. This hints at a Marangoni boat propulsion mechanism for the alginate–PEG swimmers.

**Fig. 6:**
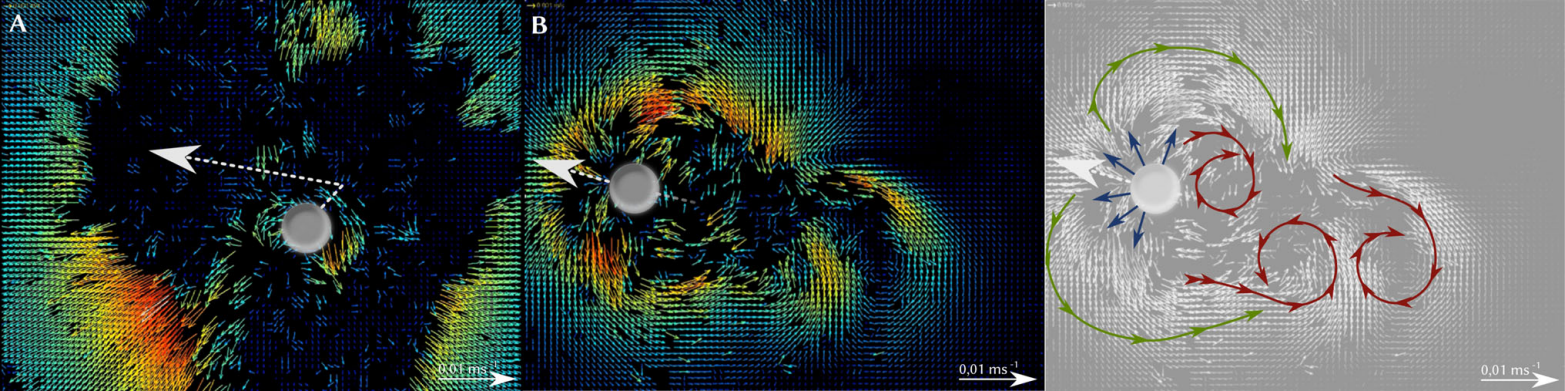
PIV measurements of a PEG–alginate swimmer **a** after dripping and **b** in motion. The velocity scale bar refers to the fluid velocities, which are also color-coded for velocity. The white dashed arrows indicate the direction of the particle motion. On the right, we indicate the fluid motion in **b** featuring radial Marangoni flow (blue), combined with flows corresponding to two counter-rotating vortices created by particle motion (green), and downstream Karman-like vortices (red)

There is further experimental evidence supporting the Marangoni boat mechanism: (a) Sinking capsules stop swimming which excludes a phoretic or Marangoni mechanism based only on the swimmer–liquid interface such as the active liquid droplet mechanisms [16, 17]. (b) Only water-soluble spreading molecules lead to prolonged propulsion because they allow regeneration of the air–water interface by re-dissolving after spreading, which is crucial to establish concentration gradients at the air–water interface. (c) Local Wilhelmy plate surface tension measurements demonstrate surface tension modulations depending on the distance to a swimmer; this demonstrates that additional surfactant is emitted close to the swimmer. (d) Particle image velocimetry (PIV) measurements and selective staining with aniline show fluid motion consistent with surfactant spreading by surface tension reduction. (e) Swimming speed depends on the diffusive mass outflux. We will develop a theory for this dependence in the theoretical part of the paper, which describes the data (without free fitting parameters). (f) Repulsion and direction reversal without reorientation of the swimmer can be explained by an accumulation of surfactant emitted by the swimmer in front of the wall. This points to a motion opposite to the surfactant concentration gradient, while the direction of motion is not completely fixed relative to swimmer orientation.

More details regarding points (d) and (e) are given below. All these results suggest that the swimmer diffusively emits surfactant which reduces the surface tension. The swimmers are spherically symmetric to a good approximation and this symmetry is strongly broken by the concentration profile in the fast moving state. The only available mechanism for symmetry breaking in the moving state is by advection to the surrounding moving liquid, which selects a swimming direction spontaneously.

The results regarding curved trajectories and wall collision suggest that spherical symmetry is not perfect and large pores in the capsule shell can select a weakly preferred propulsion direction and link capsule orientation to swimming direction. This weak link can be deleted during a normal collision with a wall, when the swimmer reverses direction without changing orientation.

#### PIV measurements

PIV measurements were performed with polymethyl methacrylate (PMMA) tracer particles with sizes between 30 and $50\,\mathrm{nm}$ and visualize the fluid flow close to the air–water interface. Figure 6 shows the results directly after swimmer synthesis by dripping (A), i.e., in the initial starting phase of the swimmer and (B) shortly after the swimming started.

In Fig. 6a, we observe strong radial spreading of surfactant by initial Marangoni flows. Then, the symmetry is spontaneously broken when swimming is initiated and Fig. 6b shows the fluid surface flow in the initial swimming stage. During swimming, we still observe radial Marangoni flows (blue) but the fluid flow around the swimming object creates a tangential backward component (green) because two counter-rotating vortices form; moreover, Karman-like vortices appear on the rear side (red). Vortex formation demonstrates that the fluid motion happens at moderate Reynolds numbers $\mathrm{Re} \sim 60$. Similar vortex structures have also been observed in Ref. [29] for disks propelled by alcohol. Nevertheless, Reynolds numbers are moderate ($\mathrm{Re} \ll 200$) such that we can expect a steady fluid flow (eventually with boundary layer separation from the sphere and stationary Marangoni vortices). Only at higher Reynolds numbers $\mathrm{Re} > 200$, we expect unsteady or even turbulent flow around a sphere [48].

**Fig. 7:**
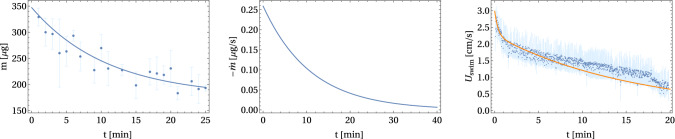
Left: Mass of the PEG–alginate swimmers as a function of time with an exponential fit $m(t) = m_\infty + m_0\exp (-t/\tau _m)$ (see text); error bars (light blue) denote the standard deviation. Middle: Resulting mass loss $-\dot{m}$ as a function of time. Right: Corresponding velocity of the swimmer averaged over 10 swimmers; error bars (light blue) denote the standard deviation. The orange line is a fit $U_\mathrm{swim}(t) = u_0\exp (-t/\tau _{u,0})+ u_1\exp (-t/\tau _{u,1})$ (see text) motivated by the existence of two swimming phases

#### Mass outflux and velocity measurements

We propose that the swimming motion is caused by surfactant that is diffusively emitted by the PEG–alginate capsule. Therefore, a relative slow reduction of the total mass of the PEG–alginate capsules should be measured, which also correlates with the swimming speed. Overall spherical symmetry of the capsule implies that the emission current density $\alpha$ is uniform on the capsule surface.

Quantitative measurements of the mass outflux are difficult. In Ref. [28] this has been achieved only indirectly by measuring the increase in surfactant in the surrounding solution. Here, we measure the mass outflux directly by removing swimmers (prepared according to the same protocol) after times $t=1,2,3,... \mathrm{min}$ from the swimming solution, dry freezing the swimmers to completely remove water from the alginate hydrogel, and determine their weight, which gives the mass *m*(*t*) of the swimmer at times $t=1,2,3,... \mathrm{min}$. Figure 7(left) shows the results for the mass averaged over 10 swimmers, error bars are the standard deviation.

Diffusive outflux through a porous shell of thickness *h* ($h<a$) approximately follows an exponential decay. The emission current density is $\alpha \approx -D_s (c_i-c_0)/h$, where $D_s$ is the diffusion constant in the gel, $c_i$ the interior PEG concentration and $c_0$ the exterior PEG concentration in solution. We assume that PEG has to diffuse over a fixed distance *h* for release; more refined release models use a time-dependent diffusion distance [28, 49, 50]. For a half-sphere, this results in $\dot{c}_i = 3\alpha /a = -3 D_s (c_i-c_0)/ha$ and an exponential decay of $c_i(t)$ and, thus, *m*(*t*). This motivates an error-weighted least-square fit with an exponential

1 $$\begin{aligned} m(t) = m_\infty + m_0\exp (-t/\tau _m), \end{aligned}$$

which describes the data well with an “empty” mass $m_\infty \approx 180\,{\upmu \mathrm{g}}$, a mass loss $m_0\approx 182\, \mathrm{mu g}$, and a time constant $\tau _m \approx 9.95\,\mathrm{min}$ [see Fig. 7(left)]. This confirms a slow diffusional surfactant release, i.e., $\alpha$ is changing on a large time scale $\tau _m$; this time scale is large compared to any microscopic time scale of the fluid flow and the surfactant diffusion. Therefore, we expect that fluid flow and surfactant concentration are always in a quasi-stationary state during swimming, i.e., adiabatically following a slowly changing $\alpha$. Differentiating with respect to time gives the mass outflux $\dot{m}$ as a function of time [see Fig. 7(middle)].

The corresponding swimming velocity $U_\mathrm{swim}$ of the PEG–alginate swimmers is measured via the single-particle tracking analysis. Figure 7(right) shows the results for the velocity averaged over 10 swimmers prepared according to the same protocol as for the mass measurements, error bars are the standard deviation. The data clearly shows a fast initial drop of the velocity in a first phase, followed by a slower decay in a second phase. During the first phase, slow diffusion of surfactant through the porous alginate matrix might not be necessary yet and gelation of the capsule alginate shell might also be incomplete. The existence of several swimming phases has also been observed for camphor disks in Ref. [27]. The second phase should be characteristic for propulsion triggered by slow diffusional release as described above. This motivates a fit

2 $$\begin{aligned} U_\mathrm{swim}(t) = u_0\exp (-t/\tau _{u,0})+ u_1\exp (-t/\tau _{u,1}) \end{aligned}$$

with two exponentials. The resulting error-weighted least-square fit describes the data well as shown in Fig. 7(right). The first phase has a time constant $\tau _{u,0} \simeq 0.37\,\mathrm{min}$ (and $u_0\simeq 0.74\,\mathrm{cm/s}$), whereas the second phase has $\tau _{u,1} \simeq 15.89\,\mathrm{min}$ (and $u_1\simeq 2.27\,\mathrm{cm/s}$), which is comparable with $\tau _m$. This further supports that diffusive release of surfactant causes the propulsion during the second phase.

## Theoretical results

### Model

In the theoretical part of the paper, we focus on the dependence of swimming speed on diffusive surfactant release. So far, this important question has not received attention in the literature. The strategy to calculate the swimming speed is as follows. We first prescribe a stationary velocity $\varvec{U} = U \varvec{e}_z$ of the swimmer and analyze the following three coupled problems for their stationary state:

(i) Surface tension reduction by surfactant adsorption at the air–water interface; depending on the volatility of the surfactant we also need to include a possible evaporation of surfactant. PEG is not volatile but the theory will apply to the physics of the Marangoni boat mechanism in general and should also explain quantitative results on camphor boats from Ref. [28]. As opposed to PEG, camphor is a volatile surfactant which quickly evaporates from the air–water interface.
(ii) Fluid flow, which includes both the fluid flow induced by motion of the half-spherical capsule and additional surfactant-induced Marangoni flow inside the fluid.
(iii) Diffusive surfactant release from the swimmer and subsequent diffusion and advection.

Solving these three coupled problems we can obtain the Marangoni forces as a function of the prescribed velocity *U* from the surfactant concentration profile. Finally, the actual swimming velocity $U=U_\mathrm{swim}$ is determined by the force equilibrium between drag force, direct propelling Marangoni forces from the surface tension gradient along the air–water–swimmer contact line, and Marangoni flow forces, which can increase either the drag or the direct Marangoni propulsion force.

The fluid flow part (ii), the drag force, and also the Marangoni forces in the force balance strongly depend on the Reynolds number. Although the Reynolds number for the PEG–alginate swimmers is moderate ($\mathrm{Re} \sim 60$), we will first develop a low Reynolds number theory, and try to address higher Reynolds numbers afterward using phenomenological results for the Nusselt number.

**Fig. 8:**
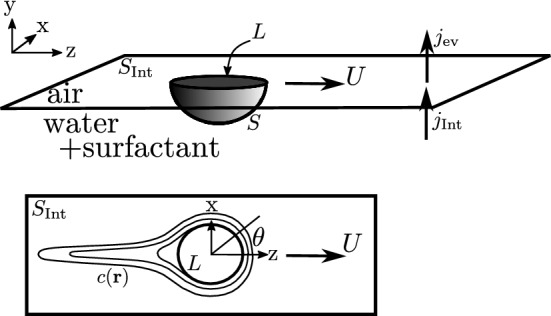
Side view (top) and top view (bottom) of the half-spherical Marangoni swimmer geometry with surfactant concentration field $c(\varvec{r})$ and coordinates

We introduce coordinates such that the origin $r=0$ is at the center of the circular planar solid surface of the half-sphere, and the liquid–air interface is at $y=0$ (with $y<0$ being the liquid phase) and $\varvec{e}_z$ will coincide with the spontaneously selected swimming direction. We also use spherical coordinates such that $\theta = 0$ is the swimming direction and the interfacial plane is located at $\phi = 0,\pi$ ($y=0$). The half-sphere has radius *a* such that the contact line is at $r=a$ and $\phi = 0,\pi$ (and parametrized by $\theta$). We denote the half-spherical surface of the swimmer by *S*, the circular air–water–swimmer contact line by *L*, and the liquid–air interface outside the swimmer as $S_\mathrm{Int}$, see Fig. 8.

#### Surface tension reduction by surfactant adsorption and evaporation

We start with problem (i), which is independent of the Reynolds number. In equilibrium, the surfactant concentration $\varGamma (\varvec{r})$ at the interface $y=0$ for a given bulk subsurface concentration $c(\varvec{r})$ is given by Langmuir adsorption

3 $$\begin{aligned} \varGamma (c) = \varGamma _\mathrm{max} {K_Lc}/(1+K_Lc) \end{aligned}$$

(with the adsorption equilibrium constant $K_L$ and the maximal surfactant surface concentration $\varGamma _\mathrm{max}$). In Langmuir adsorption, we assume ideal behavior of the surfactant molecules. According to the Gibbs adsorption isotherm, the interfacial surface tension $\gamma$ is related by $d\gamma = - RT \varGamma d\ln c$ to surface concentration and bulk concentration [51]. Together with the Langmuir equation, this leads to the Szyszkowski equation

4 $$\begin{aligned} \varDelta \gamma&= -RT\varGamma (c_0) \frac{1}{c_0} \varDelta c = -R T \varGamma _\mathrm{max}\frac{ K_L}{1+K_Lc_0}\varDelta c \end{aligned}$$

(with the gas constant $R=N_A k_B$ and $\varGamma _\mathrm{max}$ in mol per area), formulated for small local variations around a background, $c(\varvec{r}) = c_0 + \varDelta c(\varvec{r})$. Small surfactant concentration variations thus linearly reduce the surface tension,

5 $$\begin{aligned} \varDelta \gamma (\varvec{r})&= -\kappa \varDelta c(\varvec{r}) ~~\text{ with } ~~ \kappa = RT \varGamma _\mathrm{max} \frac{K_L}{1+K_Lc_0}, \end{aligned}$$

where $\varvec{r}$ is an interfacial vector with $y=0$. We choose the background $c_0$ as the bulk value $c_0 = c(\infty )$ for $|\varvec{r}|\rightarrow \infty$.

In formulating Eq. (5) locally, we already assumed that the on and off kinetics of surfactant to the interface is fast such that equilibrium can be assumed to be established instantaneously at *every* point $\varvec{r}$ on the interface. Then, also the surface concentration $\varGamma (\varvec{r})$ is slaved to the bulk and only a passive “reporter” of the bulk subsurface concentration $\left. c(\varvec{r})\right| _{y=0}$, and we do not have to solve a separate dynamics for $\varGamma (\varvec{r})$ in the interface. This assumption is typically valid for small surfactant molecules [52], in particular for water-soluble spreading molecules such as PEG. The assumption also implies that there is no flux imbalance within the interface, and also the bulk diffusive flux to the interface $S_\mathrm{Int}$ has to vanish,

6 $$\begin{aligned} j_\mathrm{Int} = -\left. D \varvec{\nabla } c(\varvec{r})\cdot \varvec{n}^\mathrm{out}\right| _{y=0} =-D \left. \partial _y c(\varvec{r})\right| _{y=0}= 0, \end{aligned}$$

which provides the corresponding boundary condition to the diffusion–advection problem (iv) in the bulk. Here, *D* is the surfactant diffusion constant in the bulk liquid. The surface concentration $\varGamma (\varvec{r})$ should also be small enough to avoid saturation of the air–water interface, which also requires water-soluble spreading molecules such as PEG.

So far, we did not consider the possibility of surfactant evaporation from the interface. This enters the balance of fluxes to and from the interface (see Fig. 8), and we have to replace Eq. (6) by

7 $$\begin{aligned} j_\mathrm{Int} = -\left. D \varvec{\nabla } c(\varvec{r})\cdot \varvec{n}^\mathrm{out}\right| _{y=0} = - j_\mathrm{ev} = k\left. c(\varvec{r})\right| _{y=0}, \end{aligned}$$

where *k* is the rate constant for evaporation.

#### Fluid flow at low Reynolds numbers

We consider the rest frame of the swimmer and linearly decompose the total fluid flow field into a field $\varvec{v}(\varvec{r})$, which is the flow field of a half-sphere pulled with velocity $U\varvec{e}_z$ through the liquid and a correction $\varvec{{v}}_\mathrm{M}(\varvec{r})$ from Marangoni flows, $\varvec{v}_\mathrm{tot}(\varvec{r}) = \varvec{v}(\varvec{r}) + \varvec{{v}}_\mathrm{M}(\varvec{r})$. For low Reynolds numbers, *both* $\varvec{v}(\varvec{r})$ and $\varvec{{v}}_\mathrm{M}(\varvec{r})$ (and the associated pressure fields) fulfill the incompressibility condition $\varvec{\nabla }\cdot \varvec{v} = 0$ and the linear Stokes equation $\mu \varvec{\nabla }^2 \varvec{v} = \varvec{\nabla } p$, where $\mu$ is the fluid viscosity. The Stokes equations for $\varvec{v}(\varvec{r})$ and $\varvec{v}_\mathrm{M}(\varvec{r})$ are decoupled because of linearity; this will be different at high Reynolds numbers.

The flow field $\varvec{v}(\varvec{r})$ of an externally pulled half-sphere has no-slip boundary conditions on the surface of the sphere, stress-free boundary conditions at the liquid–air interface, and $\varvec{v}(\infty ) = - U\varvec{e}_z$ at infinity. The total flow field $\varvec{v}_\mathrm{tot}(\varvec{r})$ also has no-slip boundary conditions on the surface of the sphere, assumes $\varvec{v}_\mathrm{tot}(\infty ) = - U\varvec{e}_z$ at infinity, but is subject to Marangoni stresses at the liquid–air interface. Consequently, the difference $\varvec{v}_\mathrm{M}(\varvec{r}) = \varvec{v}_\mathrm{tot}(\varvec{r})- \varvec{v}(\varvec{r})$ from Marangoni flows has no-slip boundary conditions on the surface of the sphere, has vanishing velocity $\varvec{v}_\mathrm{M}(\infty ) = 0$ at infinity, and is subject to Marangoni stresses at the liquid–air interface. Moreover, for all three flow fields, there is no normal flow across the liquid–air interface $\left. v_\mathrm{tot, y}(\varvec{r}) \right| _{y=0} = \left. v_y(\varvec{r}) \right| _{y=0} = \left. v_\mathrm{M, y}(\varvec{r}) \right| _{y=0} = 0$. We will assume that the liquid–air interface remains flat, even if the sphere moves. This requires that typical viscous forces remain small compared to interfacial stress, $\mu U \ll \gamma$, which is fulfilled with $\mu U \sim 10^{-5}\, \mathrm{N/m}$ for $U\sim 1 \,\mathrm{cm/s}$ and $\gamma \sim 0.07 \,\mathrm{N/m}$ for the air–water interface. We also neglect a possible curvature of the interface from wetting effects.

The Marangoni flow is caused by tangential Marangoni stresses at the liquid–air interface $y=0$,

8 $$\begin{aligned} \mu \varvec{n}^\mathrm{out}\cdot \varvec{\nabla } \left. \varvec{v}_\mathrm{M}(\varvec{r})\right| _{y=0} = \mu \partial _y \left. \varvec{v}_\mathrm{M}(\varvec{r})\right| _{y=0} = \varvec{\nabla }_S \varDelta \gamma (\varvec{r}), \end{aligned}$$

which act both on $\varvec{v}_\mathrm{M}$ and $\varvec{v}_\mathrm{tot}$.

At low Reynolds numbers, the flow field $\varvec{v}(\varvec{r})$ is given by “half” ($y<0$) the Stokes flow field around a sphere, which automatically fulfills the boundary condition $\left. v_y(\varvec{r}) \right| _{y=0} = 0$ for symmetry reasons. In spherical coordinates, the axisymmetric Stokes flow field is

9a $$\begin{aligned} \varvec{v}(\varvec{r})&= U \cos \theta u(r/a)\varvec{e}_r + U \sin \theta v(r/a) \varvec{e}_\theta ~~\text{ with } \nonumber \\ u(r/a)&= \left[ -\frac{1}{2} \left( \frac{a}{r}\right) ^3 + \frac{3}{2} \frac{a}{r} - 1 \right] \end{aligned}$$

9b $$\begin{aligned} v(r/a)&= \left[ -\frac{1}{4} \left( \frac{a}{r}\right) ^3 - \frac{3}{4} \frac{a}{r} + 1 \right] . \end{aligned}$$

#### Surfactant diffusion and advection

Surfactant molecules are emitted from the half-spherical surface *S* and diffuse into the liquid phase. At the same time, they are advected by the total fluid flow. In the stationary limit, the bulk concentration field is governed by the diffusion–advection equation

10 $$\begin{aligned} 0= \partial _t c&= D \varvec{\nabla }^2 c - (\varvec{v}(\varvec{r})+ \varvec{v}_\mathrm{M}(\varvec{r})) \cdot \varvec{\nabla } c. \end{aligned}$$

Because of the slow diffusional surfactant release the appropriate boundary condition on *S* is a constant flux boundary condition,

11 $$\begin{aligned} \left. \varvec{j}\cdot \varvec{n}\right| _S&=- D\left. \varvec{\nabla } c\cdot \varvec{n} \right| _S =\alpha , \end{aligned}$$

together with $c(\infty )=0$ and the no-flux boundary condition (6) at the interface $S_\mathrm{Int}$. The flux $\alpha$ is only slowly changing (on the time scale $\tau _m$) and approximated as a constant for the calculation of quasi-stationary fluid flow and concentration fields.

#### Drag and Marangoni forces at low Reynolds numbers

The half-spherical swimmer moving at velocity *U* is subject to three forces. First, there is the drag force, which is, at low Reynolds numbers, given by the Stokes drag for a half-sphere,

12 $$\begin{aligned} \varvec{F}_\mathrm{D} = F_\mathrm{D}\varvec{e}_z = -3\pi \mu a U \varvec{e}_z. \end{aligned}$$

Second, there is the direct Marangoni propulsion force $\varvec{F}_\mathrm{M} = F_\mathrm{M} \varvec{e}_z$ from integrating the surface stress $\varDelta \gamma (\varvec{r})= -\kappa c(\varvec{r})$ along the air–water–swimmer contact line,

13 $$\begin{aligned} F_\mathrm{M}&\equiv -\kappa \oint _L ds (\varvec{e}_n\cdot \varvec{e}_z) c(\varvec{r}) \nonumber \\&= - 2 \kappa a \int _0^\pi d\theta \cos \theta c(a,\theta )|_{y=0}. \end{aligned}$$

Third, there is the Marangoni flow force $\varvec{F}_\mathrm{M, fl} = F_\mathrm{M, fl} \varvec{e}_z$, which is by definition the force transmitted by fluid stresses of the Marangoni flow onto the sphere,

14 $$\begin{aligned} F_\mathrm{M, fl} \equiv - \int _{S} da_i \sigma _\mathrm{M, iz}. \end{aligned}$$

For low Reynolds numbers, we can apply the reciprocal theorem to the flow fields $\varvec{v}$ and $\varvec{v}_\mathrm{M}$ and their associated stress tensors to calculate the Marangoni flow force without explicitly calculating the Marangoni flow $\varvec{v}_\mathrm{M}$, as has been shown in detail in Ref. [53]. This gives the identity $0= \int _{S+S_\mathrm{Int}} da_i v_{j} \sigma _\mathrm{M, ij}$, which leads to a Marangoni flow force

15 $$\begin{aligned} F_\mathrm{M,fl}&= -\kappa \int _{S_\mathrm{Int}} dS \frac{\varvec{v}(\varvec{r})+U\varvec{e}_z}{U} \cdot \varvec{\nabla }_S c(\varvec{r}). \end{aligned}$$

The total Marangoni force

16 $$\begin{aligned} F_\mathrm{M,tot} \equiv F_\mathrm{M} + F_\mathrm{M,fl}, \end{aligned}$$

is obtained by using (13) and the Gauss theorem,

17 $$\begin{aligned} F_\mathrm{M,tot}&= \kappa \int _{S_\mathrm{Int}} dS \left( \varvec{\nabla }_S\cdot \frac{\varvec{v}(\varvec{r})}{U}\right) c(\varvec{r})\nonumber \\&=-\frac{3\kappa a}{2} \int _1^\infty d\rho \int _0^\pi d\theta \cos \theta \left( \frac{1}{\rho } - \frac{1}{\rho ^{3}} \right) {c}(\rho a,\theta )|_{y=0}. \end{aligned}$$

Because $\rho ^{-1} - \rho ^{-3} >0$ for $\rho >1$, the total Marangoni force is always positive for concentration profiles, which are increasing toward the rear side. This implies that the total Marangoni force is always propulsive, i.e., points in the same direction as the imposed velocity *U* regardless of its absolute value. This is a necessary condition for self-propulsion. When the particle is pulled by an external force, this also implies that the total Marangoni force will always support the pulling force instead of increasing the drag. The Marangoni flow contribution $F_\mathrm{M,fl}$, however, can have both signs. For $F_\mathrm{M,fl}>0$, the flow force increases the direct Marangoni force resulting in $F_\mathrm{M,tot}> F_\mathrm{M}$; for $F_\mathrm{M,fl}<0$, the flow force is directed backward and increases the drag force resulting in $F_\mathrm{M,tot}< F_\mathrm{M}$. As opposed to Ref. [38], we will find that both cases are possible.

In the stationary swimming state, drag and total Marangoni force have to be balanced,

18 $$\begin{aligned} 0= F_\mathrm{D} + F_\mathrm{M, tot} = F_\mathrm{D} + F_\mathrm{M} + F_\mathrm{M,fl}, \end{aligned}$$

such that the swimmer is force-free. This is the swimming condition that finally determines the actual swimmer velocity $U = U_\mathrm{swim}$.

### Non-dimensionalization

To proceed, we make the system of coupled equations governing our sub-problems (i)–(iii) and the Marangoni forces dimensionless by measuring lengths in units of *a*, velocities in units of *D*/*a*, concentrations in units of $\alpha a/D$, and forces in units of $D\mu$. We introduce

19 $$\begin{aligned} \varvec{\rho }&\equiv \varvec{r}/a, ~~\bar{\varvec{\nabla }} \equiv a \varvec{\nabla } = \varvec{\nabla }_\rho , ~~\bar{\varvec{v}}\equiv \varvec{v} \frac{a}{D},~~ \bar{U} \equiv U \frac{a}{D}, \nonumber \\ \bar{c}&\equiv c \frac{D}{\alpha a},~~ \bar{j} \equiv j\frac{1}{\alpha }, \nonumber \\ \bar{F}&\equiv F\frac{1}{D\mu },~~ \bar{p} \equiv p \frac{a^2}{D\mu }. \end{aligned}$$

The prescribed dimensionless velocity $\bar{U}$ of the swimmer is the first control parameter of the problem,^1^ which is related to the Reynolds number, $\mathrm{Re} = 2\bar{U}/\mathrm{Sc}$, via the Schmidt number $\mathrm{Sc} \equiv \mu /\rho D$.

Our dimensionless set of equations for problems (i)–(iii) becomes

20a $$\begin{aligned} \mathrm{(i)}&-\left. \bar{\varvec{\nabla }} \bar{c}(\varvec{\rho })\cdot \varvec{n}^\mathrm{out} \right| _{\bar{y}=0}\approx 0\nonumber \\&\text{ without } \text{ evaporation, } \end{aligned}$$

20b $$\begin{aligned}&-\left. \bar{\varvec{\nabla }} \bar{c}(\varvec{\rho })\cdot \varvec{n}^\mathrm{out} \right| _{\bar{y}=0} \approx \bar{k}\left. \bar{c}(\varvec{\rho }) \right| _{\bar{y}=0} \nonumber \\&\text{ with } \text{ evaporation, } \end{aligned}$$

20c $$\begin{aligned} \mathrm{(ii)}&\bar{\varvec{v}}_\mathrm{tot}(\varvec{\rho })&= \bar{\varvec{v}}(\varvec{\rho }) + \bar{\varvec{v}}_\mathrm{M}(\varvec{\rho }), \nonumber \\ \mathrm{(iia)}&\bar{\varvec{v}}(\rho ,\theta )&= \bar{U}\cos \theta u(\rho ) \varvec{e}_r + \bar{U} \sin \theta v(\rho ) \varvec{e}_\theta \nonumber \\&\text{ Stokes } \text{ flow } \text{ field, } \end{aligned}$$

20d $$\begin{aligned} \mathrm{(iib)}&\bar{\varvec{\nabla }}\cdot \bar{\varvec{v}}_\mathrm{M}&= 0\nonumber \\&\text{ Marangoni } \text{ flow } \text{ field, } \nonumber \\&\bar{\varvec{\nabla }}^2 \bar{\varvec{v}}_\mathrm{M}&= \bar{\varvec{\nabla }} \bar{p}_\mathrm{M}, \nonumber \\&\bar{\varvec{v}}_\mathrm{M}(\infty )&=0, \nonumber \\&\left. \bar{\varvec{v}}_\mathrm{M}(\varvec{\rho }) \right| _{\rho =1}&=0, \nonumber \\&\left. \bar{v}_\mathrm{M, y}(\varvec{\rho }) \right| _{\bar{y}=0}&=0, \nonumber \\&\left. \partial _{\bar{y}} \bar{\varvec{v}}_\mathrm{M}(\varvec{\rho }) \right| _{\bar{y}=0}&= -\mathrm{Pe} \left. \bar{\varvec{\nabla }}_{S} \bar{c}(\varvec{\rho }) \right| _{\bar{y}=0}, \end{aligned}$$

20e $$\begin{aligned} \mathrm{(iii)}&0&= \bar{\varvec{\nabla }}^2 \bar{c} - (\bar{\varvec{v}}(\varvec{\rho })+ \bar{\varvec{v}}_\mathrm{M}(\varvec{\rho })) \cdot \bar{\varvec{\nabla }} \bar{c}, \nonumber \\&\bar{c}(\infty )&= 0, \nonumber \\&\left. \bar{\varvec{j}}\cdot \varvec{n}\right| _S&=- \left. \bar{\varvec{\nabla }} \bar{c}\cdot \varvec{n} \right| _S =1, \end{aligned}$$

with the dimensionless Peclet number

21 $$\begin{aligned} \mathrm{Pe}&\equiv \frac{\kappa \alpha a^2}{D^2 \mu }= \frac{\kappa \dot{m}}{2\pi D^2 \mu }, \end{aligned}$$

where $\dot{m}= 2\pi a^2\alpha$ is the mass loss per time of the swimmer (see Fig. 7). The Peclet number is a dimensionless measure of propulsion strength.

Typical values for the PEG–alginate swimmer are very high, $\mathrm{Pe} \sim 10^7$ (see Table 2). We also introduced the dimensionless Biot number

22 $$\begin{aligned} \bar{k} \equiv \frac{ak}{D} \end{aligned}$$

governing possible evaporation, which is practically absent for PEG. From Eq. (20d), we see that the Peclet number $\mathrm{Pe}$ determines the velocity scale of the Marangoni flow field. Therefore, we can also assign a Reynolds number $\mathrm{Re}_\mathrm{M} = {2\mathrm{Pe}}/{\mathrm{Sc}}= \mathrm{Re} \mathrm{Pe}/\bar{U}$ to the Marangoni flow. Typical values for the PEG–alginate swimmer are $\mathrm{Re}_\mathrm{M} \sim 10^4$ (see Table 2), which agrees with the experimentally observed turbulent features of Marangoni flows (see Fig. 6). Via the advection with $\bar{\varvec{v}}(\varvec{\rho })+ \bar{\varvec{v}}_\mathrm{M}(\varvec{\rho })$, the concentration field $c(\varvec{\rho })$ depends both on the dimensionless velocity scale $\bar{U}$ of the Stokes field and the dimensionless velocity scale $\mathrm{Pe}$ of the Marangoni flow field, in general. All dimensionless parameters are summarized in Table 2, along with typical values for the PEG–alginate swimmers and in comparison with camphor boats according to Ref. [28].

**Table 2:** Dimensionless parameters. $\mathrm{Re}$ or $\bar{U}$, $\mathrm{Sc}$, $\mathrm{Pe}$ and $\bar{k}$ are control parameters of the problem. $\mathrm{Re}_\mathrm{M}$ and $\mathrm{Nu}$ cannot be independently controlled but characterize the resulting solutions; the swimming velocity $\bar{U}_\mathrm{swim}$ is determined by the force balance swimming condition

Parameter	Formula	Eq	PEG–alginate swimmer	Camphor boat[28]
Reynolds number $\mathrm{Re}$	$={2\rho U a}/{\mu } = {2\bar{U}}/{\mathrm{Sc}}$		$30-80$	$60-3000$
dimensionless velocity $\bar{U}$	$= U{a}/{D}$	(19)	$4\times 10^4-1.2\times 10^5$	$4\times 10^4-1.2\times 10^6$
Schmidt number $\mathrm{Sc}$	$= {\mu }/{\rho D}$		2860	1390
Peclet number $\mathrm{Pe}$	$= {\kappa \alpha a^2}/{D^2 \mu }$	(21)	$3.5\times 10^{6} - 8.8\times 10^{7}$	$(9.3\times 10^{9})(a/4\mathrm{mm})^2$
Biot number $\bar{k}$	$= {ak}/{D}$	(22)	$\approx 0$	$\approx 550$
swimming velocity $\bar{U}_\mathrm{swim}$	$= U_\mathrm{swim}{a}/{D}$	(25)	$4\times 10^4-1.2\times 10^5$	$4\times 10^4-1.2\times 10^6$
Marangoni Reynolds number $\mathrm{Re}_\mathrm{M}$	$= {2\mathrm{Pe}}/{\mathrm{Sc}}= \mathrm{Re} \mathrm{Pe}/\bar{U}$		$2.4\times 10^3-6.2\times 10^4$	$(1.4\times 10^7) (a/4\mathrm{mm})^2$
Nusselt (Sherwood) number $\mathrm{Nu}$ ($\mathrm{Sh}$)	$={-\partial _\rho \bar{c}_0(1)}/{\bar{c}_0(1)}$	(26)	(27)	

In the following, we will solve the problems (i)–(iii), in order to obtain the dimensionless direct and total Marangoni forces [see Eqs. (13) and (17)] from the concentration profiles by

23 $$\begin{aligned} \bar{F}_\mathrm{M}&= - 2 \mathrm{Pe} \int _0^\pi d\theta \cos \theta \bar{c}(1,\theta )|_{\bar{y}=0}, \end{aligned}$$

24 $$\begin{aligned} \bar{F}_\mathrm{M,tot}&=-\frac{3\mathrm{Pe}}{2} \int _1^\infty d\rho \int _0^\pi d\theta \cos \theta \left( \frac{1}{\rho } - \frac{1}{\rho ^{3}} \right) \bar{c}(\rho ,\theta )|_{\bar{y}=0}. \end{aligned}$$

for a prescribed swimmer velocity $\bar{U}$. Finally, force balance gives the dimensionless version of the swimming condition (18),

25 $$\begin{aligned} -\bar{F}_\mathrm{D} = 3\pi \bar{U}_\mathrm{swim} = \bar{F}_\mathrm{M}(\mathrm{Pe},\bar{U}_\mathrm{swim}) + \bar{F}_\mathrm{M,fl} (\mathrm{Pe},\bar{U}_\mathrm{swim}), \end{aligned}$$

which then selects the actual swimmer velocity $\bar{U}=\bar{U}_\mathrm{swim}$ as a function of the remaining control parameters $\mathrm{Pe}$ (“fuel” emission) and eventually $\bar{k}$ (evaporation).

The non-dimensionalization reveals that the coupled problems (i)–(iii) and the Marangoni forces depend on three dimensionless control parameters (see also Table 2): First, the prescribed dimensionless velocity of the swimmer $\bar{U}$; second, the Peclet number $\mathrm Pe$ characterizing the strength $\alpha$ of the surfactant emission, and third, the Biot number $\bar{k}$ characterizing the evaporation. We also see that the Peclet number both controls the strength of the Marangoni flow via Eq. (20d) and the strength of all Marangoni forces. We note, however, that $\bar{F}_\mathrm{M}/\mathrm{Pe}$ and $\bar{F}_\mathrm{M,tot}/\mathrm{Pe}$ still depend on $\bar{U}$ and $\mathrm{Pe}$ via the dependence of $\bar{c}(\varvec{\rho })$ on these parameters.

Another important finding from non-dimensionalization is that the diffusion–advection problem decouples from the Marangoni flow problem for $\mathrm{Pe} \ll \bar{U}$, where we can neglect $\varvec{v}_\mathrm{M}$ in the advection term. Then, the concentration profile is only determined by Stokes flow, becomes axisymmetric, and only depends on $\bar{U}$. In this limit, the Marangoni flow field need not to be calculated in order to calculate the total Marangoni force via Eq. (24).

### Numerical methods

Numerically, we only address the low Reynolds number regime. In general, we consider the problems (i)–(iii), i.e., solve the coupled diffusion–advection problem and the Marangoni flow problem for a prescribed swimmer velocity $\bar{U}$. From the solution for the concentration field, we then calculate the Marangoni forces as a function of $\bar{U}$ and $\mathrm{Pe}$ in order to finally solve the force balance swimming condition. We use an iterative finite element scheme to solve the full coupled problem; this approach is explained in detail in Appendix A.

### Low Reynolds number results

Low Reynolds numbers $\mathrm{Re}\ll 1$ are realized for $\bar{U} \ll \mathrm{Sc}/2$, which can still be much larger than unity as typical Schmidt numbers for surfactants in aqueous solutions are of the order of 1000 (see Table 2). Therefore, we have to discuss *both* the diffusive limit $\bar{U}\ll 1$ *and* the advective limit $\bar{U}\gg 1$.

#### Decoupled limit $\mathrm{Pe} \ll \bar{U}$

First, we will consider the limit $\mathrm{Pe} \ll \bar{U}$, where the diffusion–advection problem for a half-sphere with prescribed velocity *U* decouples from the Marangoni flow problem. We also focus on the case in the absence of evaporation first, as it is appropriate for the PEG–alginate swimmer.

Diffusive release from a moving emitter or from a resting emitter in a fluid flow can be characterized by the average Nusselt number (or Sherwood number Sh),

26 $$\begin{aligned} \mathrm{Nu} \equiv \frac{\int _S \varvec{j}(\varvec{r})\cdot \varvec{n}\,dA}{ (D/a) \int _S c(\varvec{r})\,dA}, \end{aligned}$$

which is the dimensionless ratio of the total emitted flux and the typical diffusive flux [57]. A Nusselt number of one is realized for purely diffusive transport, Nusselt numbers much larger than one indicate strong advective transport.

**Fig. 9:**
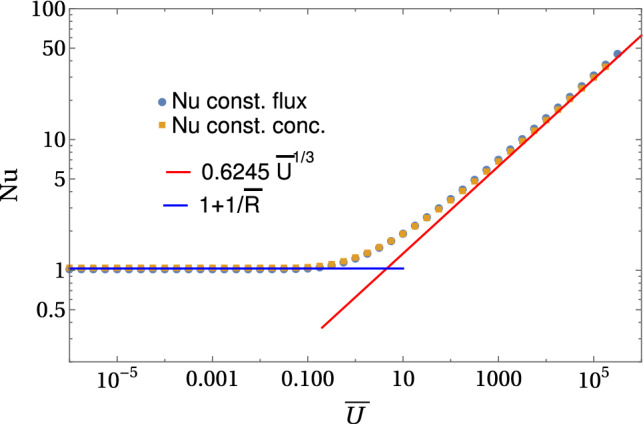
Average Nusselt number as a function of $\bar{U}$ for constant flux and constant concentration boundary conditions in the decoupled limit $\mathrm{Pe} \ll \bar{U}$. All results are from numerical FEM solutions of the axisymmetric diffusion–advection equation in two-dimensional angular representation with $\rho <\bar{R}=30$

In the decoupled limit $\mathrm{Pe} \ll \bar{U}$, we find for the Nusselt number

27 $$\begin{aligned} \mathrm{Nu} = \frac{1}{\bar{c}_0(\rho =1)}&= {\left\{ \begin{array}{ll} 1+\frac{1}{2} \tilde{U} &{} \text{ for }~\bar{U}\ll 1\\ 0.65\, \bar{U}^{1/3} &{} \text{ for }~\bar{U}\gg 1 \end{array}\right. }, \end{aligned}$$

where $c_0(\rho ) \equiv \frac{1}{2} \int _0^\pi d\theta \sin \theta \bar{c}(\rho ,\theta )$ is the zeroth Legendre coefficient. There are two regimes, a diffusive regime for $\bar{U}\ll 1$ characterized by a Nusselt number close to one and an advective regime for $\bar{U} \gg 1$ where the Nusselt number becomes large, which is also clearly supported by the numerical results in Fig. 9. The result (27) can be derived analytically [58], apart from the value of the prefactor 0.65, which we determined numerically from the data in Fig. 9. A short derivation based on scaling arguments for the advective regime is presented below. The numerical results in Fig. 9 show perfect agreement and clearly confirm the existence of just two regimes.

The Nusselt number has been originally defined for constant concentration boundary conditions $\bar{c}(1,\theta )=1$, For constant concentration boundary conditions, the result is well-known [54–57] and very similar (see Fig. 9),

28 $$\begin{aligned} \mathrm{Nu} = -\partial _\rho \bar{c}_0(\rho =1)&= {\left\{ \begin{array}{ll} 1+\frac{1}{2} \tilde{U} + ... &{} \text{ for }~\bar{U}\ll 1\\ 0.6245\, \bar{U}^{1/3} &{} \text{ for }~\bar{U}\gg 1 \end{array}\right. } \end{aligned}$$

with a prefactor that can be calculated analytically. This indicates that literature results for the Nusselt number for constant concentration boundary conditions also apply to our situation of constant flux boundary conditions, which is an important insight that we will assume to also hold for higher Reynolds numbers below.

**Fig. 10:**
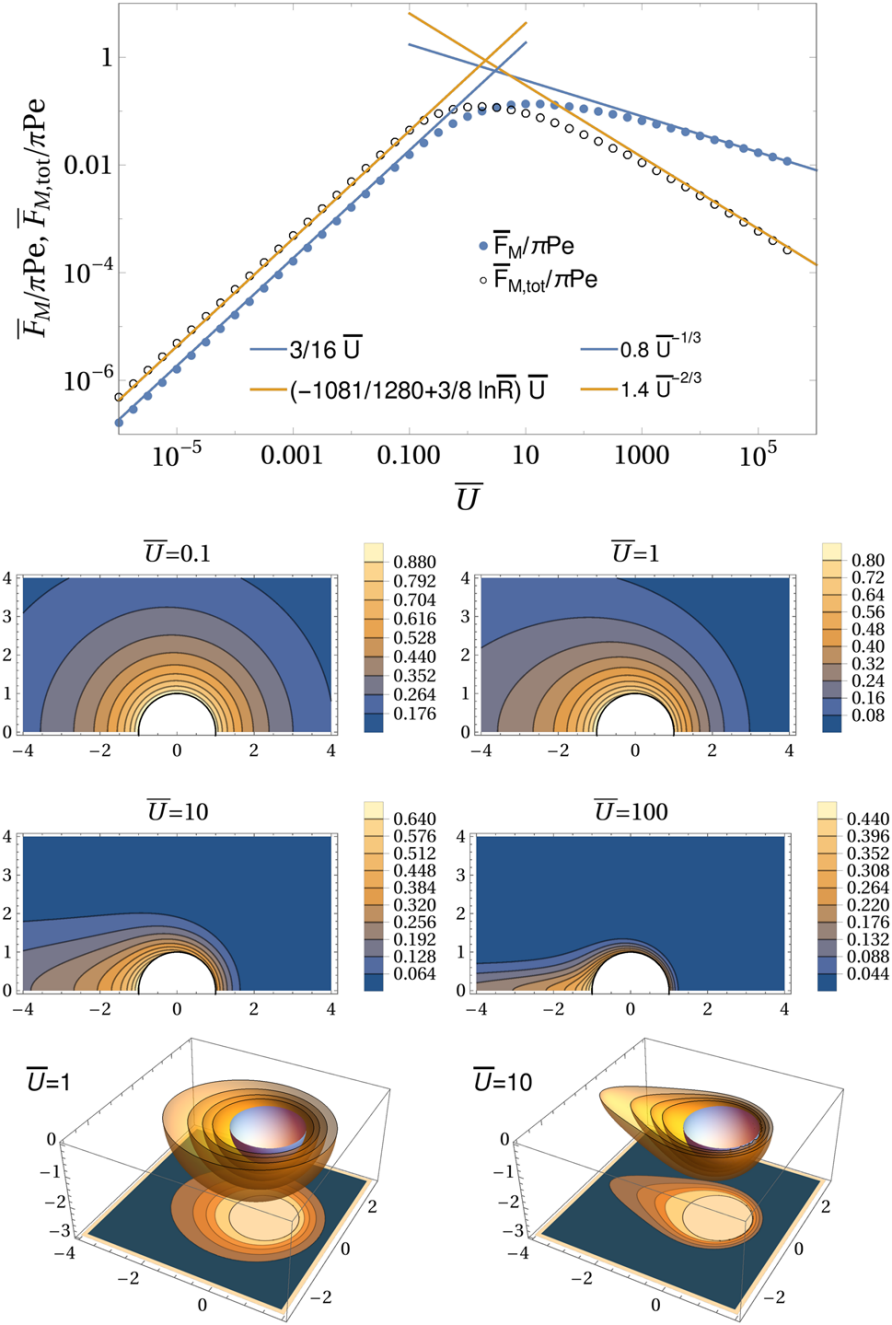
Marangoni forces ${\bar{F}_\mathrm{M}}/{\pi \mathrm Pe}$ and ${\bar{F}_\mathrm{M, tot}}/{\pi \mathrm Pe}$ as a function of imposed velocity $\bar{U}$ together with corresponding concentration profiles (in the $\bar{z}\bar{x}$-plane and in three dimensions) in the decoupled limit $\mathrm{Pe} \ll \bar{U}$. All results are from numerical FEM solutions of the axisymmetric diffusion–advection equation in two-dimensional angular representation with $\rho <\bar{R}=30$. In the advective regime $\bar{U}\gg 1$ a concentration boundary layer develops [see Eq. (31)]

In the decoupled limit $\mathrm{Pe} \ll \bar{U}$, we also find a diffusive and an advective regime for the Marangoni forces

29 $$\begin{aligned} \frac{\bar{F}_\mathrm{M} }{\pi \mathrm{Pe}}&= {\left\{ \begin{array}{ll} \frac{3}{16} \bar{U} &{} \text{ for }~\bar{U}\ll 1\\ d_\mathrm{M} \bar{U}^{-1/3}~\text{ with }~d_\mathrm{M}\simeq 0.8 &{} \text{ for }~\bar{U}\gg 1 \end{array}\right. }, \end{aligned}$$

30 $$\begin{aligned} \frac{\bar{F}_\mathrm{M, tot}}{\pi \mathrm Pe}&= {\left\{ \begin{array}{ll} - \frac{1081}{1280} \bar{U} +\frac{3}{8} \bar{U}\ln \bar{R} &{} \text{ for }~\bar{U}\ll 1\\ d_\mathrm{M, tot} \bar{U}^{-2/3}~\text{ with }~d_\mathrm{M, tot}\simeq 1.4 &{} \text{ for }~\bar{U}\gg 1 \end{array}\right. }, \end{aligned}$$

where numerical constants $d_\mathrm{M}$ and $d_\mathrm{M, tot}$ are obtained from the numerical results, see Fig. 10, and $\bar{R}$ is the radial system size. Again, the numerical results (Fig. 10) show perfect agreement and clearly confirm the existence of just two regimes, a diffusive and an advective regime. Direct and total Marangoni force reach maximal values $\bar{F}_\mathrm{M},\bar{F}_\mathrm{M, tot}\sim 0.15\pi \mathrm{Pe}$ in the crossover region $\bar{U} \sim 1$ between diffusive and advective transport.

Also, the results (29) and (30) can be derived analytically from a calculation of the concentration field [58], apart from the value of the numerical constants. Here we present a short derivation based on scaling arguments. In the diffusive limit $\bar{U}\ll 1$, there is a linear response of the concentration field, which leads to a linear response of the Nusselt number and Marangoni forces. The coefficients can be calculated by perturbation theory about the concentration field $c^{(0)}(\varvec{\rho }) = 1/\rho$ at $\bar{U}=0$ in powers of $\bar{U}$. A remarkable result of this calculation is that the linear term for the total Marangoni force diverges logarithmically with the system size *R*, see Eq. (30), while the linear term for the direct Marangoni force stays finite. This means that the Marangoni flow forces strongly *increase* the direct force for $\bar{U}\ll 1$; such a behavior could not be found in Ref. [38]. For very large system sizes $\bar{R}\gg 1/\bar{U}$, the large scale cutoff $\bar{R}$ in (30) will be replaced by $1/\bar{U}$ because the convection term can no longer be treated perturbatively in the region $\rho \gg 1/\bar{U}$, regardless how small $\bar{U}$ is [55]. We also note that the result $\mathrm{Nu} \approx 1+\frac{1}{2} \tilde{U}$ from Eqs. (27) and (28) for the Nusselt number in the diffusive regime $\bar{U}\ll 1$ is derived from the non-perturbative matching procedure for very large system sizes $\bar{R}\gg 1/\bar{U}$ [55], while a perturbative calculation gives $\mathrm{Nu} \approx 1+1/\bar{/}{R}+ O(\bar{U}^2)$ with the radial system size $\bar{R}$. This perturbative result describes our numerical data for a finite system actually better, see Fig. 9.

In the limit of strong advection $\bar{U}\gg 1$, a concentration boundary layer develops around the half-sphere, as can be clearly seen in the concentration profiles in Fig. 10). Its width $\varDelta r$ is determined by the distance that a surfactant molecule can diffuse during the time $\varDelta t\sim a/v(\varDelta r/a)$ [see Eq. (9b)] it takes to be transported along the sphere by advection: $\varDelta r^2 \sim D \varDelta t$. Because $v(\varDelta r/a) \sim U \varDelta r/a$ [see Eq. (9b)], we find

31 $$\begin{aligned} \varDelta \rho = \varDelta r/a \sim \bar{U}^{-1/3}. \end{aligned}$$

This is a classic result for the diffusion–advection problem for constant concentration boundary conditions [54, 57], but also holds for constant flux boundary conditions. Because the concentration will drop in radial direction from its value at the surface *S* of the half-sphere to zero within the concentration boundary layer of width $\varDelta \rho$, we also have $1= -\partial _\rho \bar{c}(\rho =1,\theta )\sim \bar{c}(\rho =1)/\varDelta \rho$, which leads to a scaling

32 $$\begin{aligned} \bar{c}(\rho =1,\theta ) \sim \varDelta \rho \sim \bar{U}^{-1/3} \end{aligned}$$

of the symmetry-breaking concentration level at the surface *S* of the half-sphere for constant flux boundary conditions. For strong advection, the Marangoni forces decrease as a function of $\bar{U}$ because the concentration boundary layer width $\varDelta \rho$, in which forces are generated, and the concentration level around the sphere, to which forces are proportional, both decay with velocity as $\bar{U}^{-1/3}$.

The scaling property (32) for $\bar{c}(\rho =1,\theta )$ directly explains the results (27), $\mathrm{Nu} \sim 1/\bar{c}(\rho =1,\theta ) \sim \bar{U}^{1/3}$, for the Nusselt number and (29), $\bar{F}_\mathrm{M}/\mathrm{Pe}\sim \bar{c}(\rho =1,\theta ) \sim \bar{U}^{-1/3}$, for the direct Marangoni force in the advective limit $\bar{U}\gg 1$. They are clearly confirmed by all numerical results in Figs. 9 and 10. The result for the total Marangoni force (30) seems to deviate from this advective scaling Here, the expected scaling from the radial concentration boundary layer of width $\varDelta \rho$ is $\bar{F}_\mathrm{M,tot}/\mathrm{Pe}\sim \varDelta \rho ^2 \bar{c}(\rho =1,\theta ) \sim \bar{U}^{-1}$ (see Eq. (24)), which is clearly not found in the numerics (yellow line in Fig. 10). The reason is that this contribution is actually only sub-dominant. The leading contribution comes from the advective tail of angular width $\varDelta \theta \sim \bar{U}^{-1/3}$; the width of the tail follows from the scaling of the stream function $\psi \propto r^2\varDelta \theta ^2$ in the tail and $\psi \propto 3\varDelta r^2\sin ^2\theta /2$ in the boundary layer and the fact that a fluid particle should follow a Stokes flow stream line $\psi = \mathrm{const}$ in the advective limit. Therefore, the dominant contributions in Eq. (24) are $\bar{F}_\mathrm{M,tot}\sim \mathrm{Pe} \varDelta \theta \bar{c}(\rho =1,\theta )\sim \bar{U}^{-2/3}$ in agreement with the numerical results in Fig. 10.

Comparing the curves for direct and total Marangoni force in Fig. 10, we observe a crossing such that ${\bar{F}_\mathrm{M}} < {\bar{F}_\mathrm{M, tot}}$ in the diffusive regime $\bar{U}\ll 1$, while ${\bar{F}_\mathrm{M}} > {\bar{F}_\mathrm{M, tot}}$ in the advective regime $\bar{U} \gg 1$. This means that the Marangoni flow force $\bar{F}_\mathrm{M,fl} = \bar{F}_\mathrm{M,tot} - \bar{F}_\mathrm{M}$ *increases* the propulsion force in the diffusive regime $\bar{U} \ll 1$ but *decreases* the propulsion force (or increases the drag) for $\bar{U} \gg 1$. This subtle result is related to the structure of the Marangoni flows, which are generated by the surfactant concentration gradients $\left. \bar{\varvec{\nabla }}_{S} \bar{c}(\varvec{\rho }) \right| _{\bar{y}=0}$ within the liquid–air interface (see Eq. (20d)) and can be qualitatively rationalized with the help of Eq. (15) for the Marangoni flow force.

**Fig. 11:**
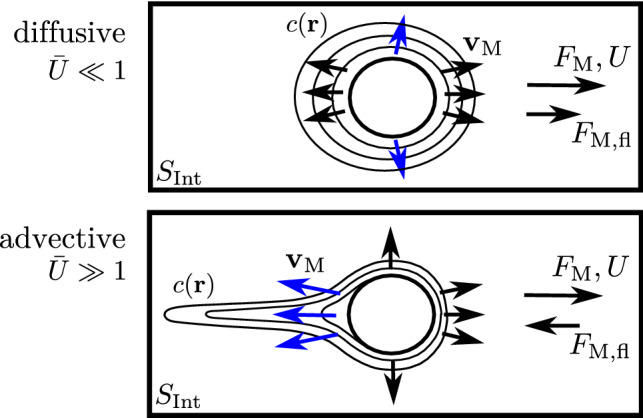
Schematic of concentration field (as concentration contour lines) and Marangoni flow field (arrows) and resulting direct force $F_\mathrm{M}$ and Marangoni flow force $F_\mathrm{M,fl}$ in the diffusive ($\bar{U}\ll 1$) and advective ($\bar{U}\gg 1$) regime. The dominant Marangoni flow contributions are marked in blue. In the diffusive regime, Marangoni flows increase the direct force, in the advective regime they decrease the direct force. It is important to note that concentration contour lines are not approaching a spherical shape close to the swimmer in the advective regime because we have constant flux boundary conditions such that the swimmer surface is a contour of constant radial gradient by construction. The elongated tail gives rise to a slower decay of the gradient itself and, thus, larger radial gradients in the tail, which is reflected by additional contour lines emerging on the rear side

We can decompose the surfactant concentration gradient into tangential and radial components

33 $$\begin{aligned} \left. \bar{\varvec{\nabla }}_{S} \bar{c}(\varvec{\rho }) \right| _{\bar{y}=0} = \frac{1}{\rho }\partial _\theta \bar{c}\, \varvec{e}_\theta + \partial _\rho \bar{c} \,\varvec{e}_r. \end{aligned}$$

Because of advection the tangential component points from the front to the rear corresponding to an increasing surfactant concentration toward the rear ($\partial _\theta \bar{c}>0$). It gives rise to a forward Marangoni flow and $F_\mathrm{M,fl}>0$ in Eq. (15) because $-\varvec{e}_z \cdot \rho ^{-1}\partial _\theta \bar{c} \varvec{e}_\theta \propto \sin \theta >0$. This is the dominating effect in the diffusive regime $\bar{U} \ll 1$, where the perturbation theory gives to leading linear order in $\bar{U}$ a contribution of the form $\bar{U}\bar{c}^{(1)} \propto -\bar{U}\bar{c}_1(\rho ) \cos \theta$ resulting in $\rho ^{-1}\partial _\theta \bar{c}= \bar{U}\rho ^{-1}\bar{c}_1(\rho ) \sin \theta >0$. The front-directed tangential Marangoni flow components give rise to the blue flow directions in the schematic in Fig. 11 (top).

The radial component points inward ($\partial _\rho \bar{c} <0$) because of the radially decaying surfactant concentration. This gives rise to radially outward Marangoni flows. Because $-\varvec{e}_z \cdot \partial _\rho \bar{c} \varvec{e}_r\propto \cos \theta$ in Eq. (15), this increases the direct force in the front (around $\theta =0$) but decreases it in the back (around $\theta =\pi$). Advection leads to bigger surfactant concentrations in the rear, which also result in bigger radial concentration gradients on the rear side and lead to an overall decrease in the direct force $\bar{F}_\mathrm{M,fl}<0$ and, thus, an increased drag. This is the dominating effect in the advective regime $\bar{U}\gg 1$, where a concentration boundary layer of width $\varDelta \rho \sim \bar{U}^{-1/3}$ forms around the half-sphere, which results in steep radial concentration gradients that are stronger on the rear side. The stronger radial Marangoni flow components in the rear are indicated by the blue arrows in Fig. 11 (bottom); this phenomenon is also visible in the experimental PIV measurements in Fig. 6b during motion. There are also slightly bigger radial concentration gradients in the diffusive regime $\bar{U}\ll 1$, but they are sub-dominant for the slow radial decay of the function $\bar{c}_1(\rho )$ in the absence of a radial concentration boundary layer.

#### Strong Marangoni flow $\mathrm{Pe} \gg \bar{U}$

For weak Marangoni flow, $\mathrm{Pe} \ll \bar{U}$, we could decouple the diffusion–advection problem and obtained to regimes, a diffusive or linear response regime for $\bar{U}\ll 1$ and an advective regime for $\bar{U}\gg 1$. Now we increase the Peclet number $\mathrm{Pe}$ and, thus, the Marangoni flow. For a strong Marangoni flow, $\mathrm{Pe} \gg \bar{U}$, the linear response regime $\bar{U}\ll 1$ becomes modified. We first have to consider the dominant Marangoni flow problem (iib), which determines the main component of the fluid flow in the diffusion–advection problem (iii). The Marangoni flow pattern is a stationary Marangoni vortex ring around the spherical swimmer below and parallel to the fluid interface $S_\mathrm{Int}$. Because this solution lacks axisymmetry an analytical solution is no longer possible. Nevertheless, we can obtain novel scaling results for concentration profile and Marangoni flow field in a concentration boundary layer of width $l_c$ below the fluid interface $S_\mathrm{Int}$ along similar lines as Refs. [59, 60].

The surfactant is emitted from the sphere with the fixed current density $\left. \bar{j}\right| _S=1$. Advection by the Marangoni flow $\bar{\varvec{v}}_\mathrm{M}$ concentrates the surfactant in the boundary layer of width $\bar{l}_c$ below $S_\mathrm{Int}$. It takes a time $t \sim r/v_\mathrm{M}$ to reach a radial distance *r*. During this time, the surfactant diffuses over a distance $l_c \sim (D t)^{1/2} \sim (Dr/v_\mathrm{M})^{1/2}$ or $\bar{l}_c \sim (\rho /\bar{v}_\mathrm{M})^{1/2}$ in vertical *y*-direction, which sets the boundary layer width $\bar{l}_c$. Because we are at low Reynolds numbers, the laminar boundary layer below the fluid interface $S_\mathrm{Int}$ is of the size $\delta \sim a$ ($\bar{\delta } \sim 1$) set by the sphere. The laminar boundary layer governs the decay of the Marangoni flow field $v_\mathrm{M}$ in *y*-direction.

Moreover, we have mass conservation of the emitted surfactant. The total advective flow $\bar{J} \sim 2\pi \bar{c} \bar{v}_\mathrm{M}\rho \bar{l}_c$ below the interface at distance $\rho$ will always equal the original flow $\bar{J}=2\pi$ that is emitted at the half-sphere,

34 $$\begin{aligned} 1 = \bar{J}/2\pi \sim \bar{c} \bar{v}_\mathrm{M}\rho \bar{l}_c \sim \bar{c} \bar{v}_\mathrm{M}^{1/2}\rho ^{3/2} . \end{aligned}$$

In addition, the Marangoni boundary condition (see Eq. (20d) gives a second equation

35 $$\begin{aligned} - \mathrm{Pe} \partial _\rho \bar{c} = \partial _{\bar{y}} \bar{v}_\mathrm{M} \sim \frac{\bar{v}_\mathrm{M}}{\bar{\delta }} \sim \bar{v}_\mathrm{M} \end{aligned}$$

for concentration and velocity, which follows from the definition of the laminar boundary layer width $\bar{\delta }$. Combining both Eqs. (34) and (35), we find a differential equation for $\bar{c}(\rho )$, which we solve with the boundary condition $\bar{c}(\infty )=0$ resulting in

36 $$\begin{aligned} \bar{c}(\rho )&= \bar{c}(1) \rho ^{-2/3} ~~\text{ with }~~ \bar{c}(1) \sim \mathrm{Pe}^{-1/3}, \end{aligned}$$

37 $$\begin{aligned} \bar{v}_\mathrm{M}&\sim \bar{c}^{-2} \rho ^{-3} \sim \bar{c}^{-2}(1) \rho ^{-5/3} \sim \mathrm{Pe}^{2/3} \rho ^{-5/3}. \end{aligned}$$

We see that the advective current $\bar{j}_\mathrm{M} \sim \bar{c} \bar{v}_\mathrm{M}\sim \mathrm{Pe}^{1/3} \rho ^{-7/3}$ becomes smaller than the corresponding diffusive current $\bar{j}_D \sim -\partial _\rho \bar{c} \sim \mathrm{Pe}^{-1/3} \rho ^{-5/3}$ for $\rho > \mathrm{Pe}$. Then, our assumption of advective transport breaks down, and this should mark the boundary of the Marangoni advection dominated region. Therefore, $\rho _\mathrm{M} \sim \mathrm{Pe}$ should be the scaling of the size of the Marangoni vortex around the sphere for low Reynolds numbers. At larger distances, a crossover to diffusive transport with $\bar{c} \propto \rho ^{-1}$ sets in.

So far, we considered the leading order of our problem by setting $\bar{U}\approx 0$; going one order further, we get the linear response for small $\bar{U}$ with the ansatz $\bar{c} = \bar{c}^{(0)} + \bar{U} \bar{c}^{(1)}$ with $\bar{c}^{(0)}(\rho )$ given by (36). In the total flow $\varvec{v} + \varvec{v}_\mathrm{M}$, the Marangoni flow (37) is the zeroth order result, $\varvec{v}_\mathrm{M}= \varvec{v}_\mathrm{M}^{(0)}$, while the Stokes swimming flow $\varvec{v}= \varvec{v}^{(1)}$ is linear in $\bar{U}$. In an advection dominated situation, the constant flux relation (34) still holds in the presence of Stokes flow,

38 $$\begin{aligned} 1 \sim (\bar{c}^{(0)} + \bar{U} \bar{c}^{(1)}) (\bar{U}\bar{u}\cos \theta + \bar{v}_\mathrm{M})^{1/2}\rho ^{3/2}, \end{aligned}$$

where $\bar{u}(\rho )$ is the radial component of the Stokes flow. Expanding up to first order in $\bar{U}$ and using (34) for the leading order, we find

39 $$\begin{aligned} \bar{c}^{(1)}(\rho ) \sim \frac{1}{ \bar{v}_\mathrm{M}^{1/2}(\rho )} \bar{c}^{(0)}(\rho ) \bar{u}(\rho ) \sim \mathrm{Pe}^{-2/3} \rho ^{1/6} \bar{u}(\rho ). \end{aligned}$$

This contribution is symmetry breaking; inserting this scaling of the concentrations into the relations (23) and (24 for the Marangoni forces, we obtain

40 $$\begin{aligned} \frac{ \bar{F}_\mathrm{M}}{\pi \mathrm{Pe}}&\sim \bar{U} \mathrm{Pe}^{-2/3},&\frac{ \bar{F}_\mathrm{M,tot}}{\pi \mathrm{Pe}}&\sim \bar{U} \mathrm{Pe}^{-2/3}. \end{aligned}$$

**Fig. 12:**
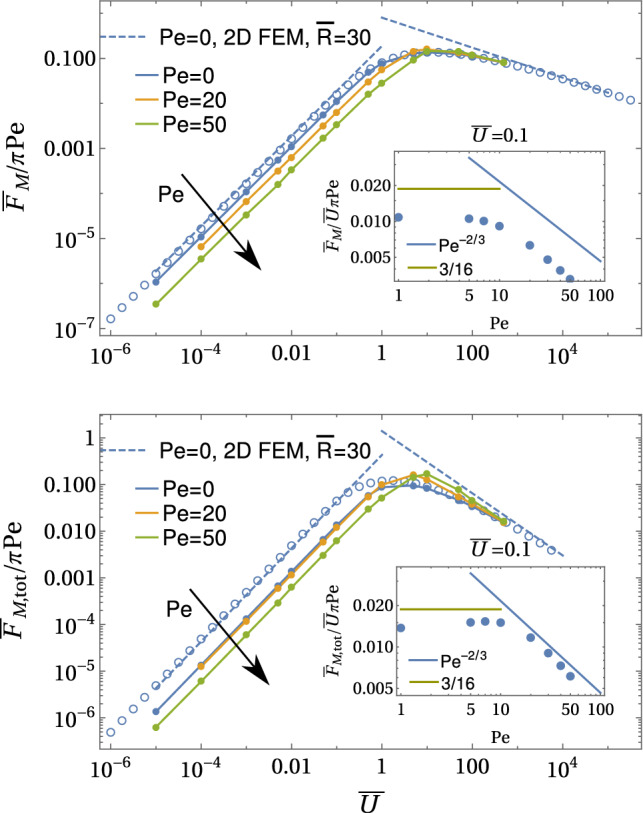
Iterative three-dimensional FEM results for ${ \bar{F}_\mathrm{M}}/\pi \mathrm{Pe}$ (top) and ${\bar{F}_\mathrm{M,tot}}/\pi \mathrm{Pe}$ (bottom) as a function of $\bar{U}$ for $\mathrm{Pe} = 0-50$ for a cubic system with $-7< \bar{y} <0$, $0<\bar{x}<7$, $-7<\bar{z}<7$. Insets show the slopes ${ \bar{F}_\mathrm{M}}/\bar{U}\pi \mathrm{Pe}$ and ${\bar{F}_\mathrm{M,tot}}/\bar{U}\pi \mathrm{Pe}$ as a function of $\mathrm{Pe}$ calculated from the results for $\bar{U}=0.1$. Artificial symmetry breaking from lattice irregularities/defects is prevented by averaging all measured quantities over two simulations with *U* and $-U$. Blue open circles are results for $\mathrm{Pe}=0$ from FEM solutions to the axisymmetric diffusion–advection equation in two-dimensional angular representation with $\bar{R}=30$. The slope in the linear response regime for $\bar{U}\ll 1$ is reduced according to Eq. (40). Results for $\bar{U}\gg 1$ are essentially not affected by strong Marangoni flows $\mathrm{Pe}\gg {\bar{U}}$

We checked these predictions numerically in Fig. 12 by using our iterative FEM approach (see Appendix A), which is possible up to $\mathrm{Pe}\sim 50$ and find good agreement, in particular, for the predictions $\bar{F}_\mathrm{M}/\mathrm{Pe} \propto \mathrm{Pe}^{-2/3}$ and $\bar{F}_\mathrm{M,tot}/\mathrm{Pe} \propto \mathrm{Pe}^{-2/3}$, which will be most important for the swimming speed relation (see insets in Fig. 12). Moreover, these numerical FEM results show that both prefactors in Eq. (40) are of order unity but hard to quantify because of finite size effects.

This shows that Marangoni flows depress the total driving force in the linear response regime by a factor $\mathrm{Pe}^{-2/3}$ reflecting the fact that it is harder to break the symmetry in the presence of the strong Marangoni flow advection. Numerical results in Fig. 12 also show that the total Marangoni force is somewhat larger than the direct Marangoni force, $\bar{F}_\mathrm{M,tot} > \bar{F}_\mathrm{M}$. In this respect, our previous results for linear response regime for $\mathrm{Pe}\ll \bar{U}$ remain unchanged: the Marangoni flow force *increases* the direct force.

In the advection-dominated regime $\bar{U}\gg 1$, on the other hand, results are essentially not affected by increasing the Peclet number $\mathrm{Pe}$ into the regime of strong Marangoni flows $\mathrm{Pe}\gg {\bar{U}}$, as the numerical results in Fig. 12 show: all curves for the Marangoni forces converge to the previous diffusion–advection results for $\bar{U} \gg 1$. The reason for this behavior is that the flow field $\varvec{v}$ will still give rise to a concentration boundary layer of thickness $\varDelta \rho \sim \bar{U}^{-1/3}$ around the sphere, which determines the concentration field and, thus, the Marangoni forces. On the scale of the boundary layer, the Marangoni flows $\varvec{v}_\mathrm{M}$ are not yet developed; they develop only further away at $1\ll \rho < \rho _\mathrm{M} \sim \mathrm{Pe}$ because of the no-slip boundary condition for the Marangoni flow in (iib). Therefore, the results for $\bar{U}\gg 1$ remain essentially unaffected by a strong Marangoni flow for $\mathrm{Pe}\gg {\bar{U}}$.

#### Evaporation

In the presence of evaporation, the boundary condition for the diffusion–advection problem changes at the air–water interface $S_\mathrm{Int}$. We then have the convective (Robin) boundary condition (20b), which is governed by the dimensionless Biot number (22), instead of the Neumann condition (20a), which is recovered for vanishing Biot number $\bar{k}=0$. In general, evaporation of surfactant depletes the interface of surfactant and, thus, decreases the Marangoni driving forces (both direct and flow force).

For volatile camphor, $k \approx \sim 10^{-4}\, \mathrm{m/s}$ has been suggested [26], which corresponds to a high Biot number $\bar{k} = ak/D \approx 550$ for the camphor disks from [28]. PEG, on the contrary, has a negligible Biot number as it is not volatile. As a consequence of the new convective boundary condition, the concentration profile will fall off exponentially perpendicular to the interface in the outward direction on a dimensionless extrapolation length scale $\varDelta \bar{y}\sim 1/\bar{k}$ given by the inverse of the Biot number.

In the presence of evaporation, we can develop a qualitative scaling theory based on the assumption that the total evaporation flux balances the total emission flux of surfactant in a stationary state, which gives

41 $$\begin{aligned} -\int _{S_\mathrm{Int}} \left. \partial _{\bar{y}} \bar{c}(\varvec{\rho })\right| _{\bar{y}=0} = 2\pi \end{aligned}$$

in dimensionless quantities and determines the derivatives $\left. \partial _{\bar{y}} \bar{c}(\varvec{\rho })\right| _{\bar{y}=0}$ at the scaling level. Via the convective boundary condition (20b), this also determines the surface concentration $\left. \bar{c}(\varvec{\rho })\right| _{\bar{y}=0}$. Moreover, the convective boundary condition should reduce to our previous results for the Neumann condition for small Biot numbers $\bar{k}$, where the evaporation flux $\bar{j}_\mathrm{ev} =\bar{k}\left. \bar{c}(\varvec{\rho })\right| _{\bar{y}=0}$ is smaller than the dominating transport flux, which is the diffusive or Marangoni flux for $\bar{U}\ll 1$ and the convective flux for $\bar{U}\gg 1$.

**Fig. 13:**
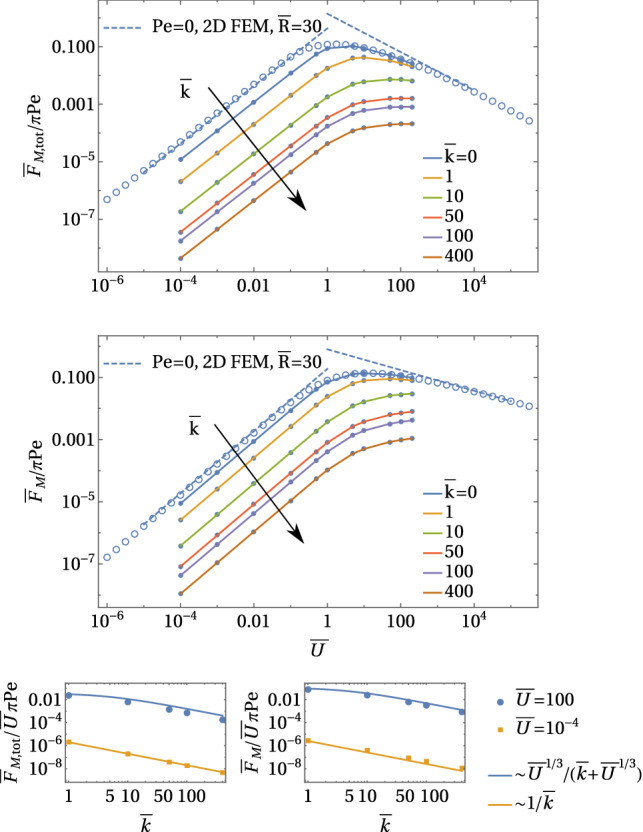
Iterative three-dimensional FEM results for ${ \bar{F}_\mathrm{M}}/\pi \mathrm{Pe}$ (top) and ${\bar{F}_\mathrm{M,tot}}/\pi \mathrm{Pe}$ (middle) as a function of $\bar{U}$ for $\mathrm{Pe} = 0$ and Biot numbers $\bar{k}=0-400$ for a half-cylindrical system with $\rho <15$, $\bar{x}>0$, $-4< \bar{y} <0$, Blue dashed lines are results for $\bar{k}=0$ from FEM solutions to the axisymmetric diffusion–advection equation in two-dimensional angular representation with $\bar{R}=30$. Forces in the diffusive regime $\bar{U}\ll 1$ are reduced according to Eq. (42). Forces in the advective regime $\bar{U}\gg 1$ are reduced according to Eq. (43). Bottom row: FEM results for ${ \bar{F}_\mathrm{M}}/\pi \mathrm{Pe}$ and ${\bar{F}_\mathrm{M,tot}}/\pi \mathrm{Pe}$ as a function of Biot number $\bar{k}$ for $\bar{U}=10^{-4},100$ in comparison with scaling results in Eqs. (42) and (43)

For the diffusion or Marangoni dominated situation for $\bar{U}\ll 1$, the concentration and, thus, evaporation is distributed over the whole interface $S_\mathrm{Int}$, i.e., there is no concentration boundary layer around the half-sphere. Therefore, flux balance (41) leads to $\left. \partial _{\bar{y}} \bar{c}(\varvec{\rho })\right| _{\bar{y}=0} \sim O(1)$ resulting in $\left. \bar{c}(\varvec{\rho })\right| _{\bar{y}=0} \sim 1/\bar{k}$ because of the convective boundary condition (20b). Then, also $\bar{F}_\mathrm{M}\sim 1/\bar{k}$ and $\bar{F}_\mathrm{M,tot}\sim 1/\bar{k}$. Moreover, the evaporation flux dominates over the diffusive or Marangoni fluxes (which are *O*(1)) only for $\bar{k}>\bar{k}_0$ with a crossover value $\bar{k}_0 =O(1)$, which determines the crossover to the non-evaporative case. Our numerical results in Fig. 13 suggest $\bar{k}_0 \ll 1$. Therefore, we expect

42 $$\begin{aligned} \bar{F}_\mathrm{M}&\sim \left. \bar{F}_\mathrm{M}\right| _{\bar{k}=0} \frac{1}{\bar{k}+\bar{k}_0},&\bar{F}_\mathrm{M,tot}&\sim \left. \bar{F}_\mathrm{M,tot}\right| _{\bar{k}=0} \frac{1}{\bar{k}+\bar{k}_0}. \end{aligned}$$

We checked these predictions numerically in Fig. 13 by using our iterative FEM approach (see Appendix A) and find good agreement. The plots in the bottom row (yellow symbols) show that the dependence on $\bar{k}$ for $\bar{U}\ll 1$ is described very well by $\bar{F}_\mathrm{M} = \left. \bar{F}_\mathrm{M}\right| _{\bar{k}=1}/\bar{k}$ and $\bar{F}_\mathrm{M,tot} = \left. \bar{F}_\mathrm{M,tot}\right| _{\bar{k}=1}/\bar{k}$ suggesting $\bar{k}_0 \ll 1$.

For the advection-dominated situation for $\bar{U}\gg 1$ the concentration and, thus, evaporation is present only in the concentration boundary layer of radial width $\varDelta \rho \sim \bar{U}^{-1/3}$ around the half-sphere and in the advection tail. Therefore, the flux balance (41) leads to $\left. \partial _{\bar{y}} \bar{c}(\varvec{\rho })\right| _{\bar{y}=0} \sim \bar{U}^{1/3}$ and $\left. \bar{c}(\varvec{\rho })\right| _{\bar{y}=0} \sim \bar{U}^{1/3}/\bar{k}$ because of the convective boundary condition (20b). Then, also $\bar{F}_\mathrm{M}\sim \bar{U}^{1/3}/\bar{k}$ and $\bar{F}_\mathrm{M,tot}\sim \bar{U}^{1/3}/\bar{k}$ if the evaporation flux dominates over the convective fluxes. The convective flux at the interface and at the boundary layer ($\rho \approx 1+\varDelta \rho$) is in radial direction $\bar{j}_u =\bar{U}\bar{u}(\rho ) \cos \theta \bar{c}(\rho ) \sim \bar{U}\varDelta \rho ^2 \bar{c}(\rho ) \sim \bar{U}^{1/3} \bar{c}(\rho )$ and $\bar{j}_v =\bar{U}\bar{v}(\rho ) \sin \theta \bar{c}(\rho ) \sim \bar{U}\varDelta \rho \sin \theta \bar{c}(\rho ) \sim \bar{U}^{2/3}\sin \theta \bar{c}(\rho )$ in $\theta$-direction. In the advection tail (of angular width $\varDelta \theta \sim \bar{U}^{-1/3}$), this also leads to $\bar{j}_v \sim \bar{U}^{1/3} \bar{c}(\rho )$. Therefore, the evaporation flux starts to dominate over the convective radial flux and the flux in $\theta$-direction in the advection tail for $\bar{k}> \bar{U}^{1/3}$; only then we see the effects of evaporation. Therefore, we expect

43 $$\begin{aligned} \bar{F}_\mathrm{M}&\sim \left. \bar{F}_\mathrm{M}\right| _{\bar{k}=0} \frac{\bar{U}^{1/3}}{\bar{k}+\bar{U}^{1/3}},&\bar{F}_\mathrm{M,tot}&\sim \left. \bar{F}_\mathrm{M,tot}\right| _{\bar{k}=0} \frac{\bar{U}^{1/3}}{\bar{k}+\bar{U}^{1/3}} \end{aligned}$$

for $\bar{U}\gg 1$. We checked these predictions numerically in Fig. 13 and find good agreement. The plots in the bottom row (blue symbols) show that the dependence on $\bar{k}$ for $\bar{U}\gg 1$ agrees very well with Eq. (43).

In summary, we see a reduction of all Marangoni forces by evaporation both in the linear response regime $\bar{U}\ll 1$ but also in the regime $\bar{U}\gg 1$ of strong advection. In both regimes, evaporation reduces the surfactant concentration, which decreases the Marangoni forces.

#### Force balance and swimming condition

Now we have a rather complete picture of the solution of problems (i)–(iii), i.e., diffusion–advection coupled to hydrodynamics for a prescribed swimmer velocity $\bar{U}$ at low Reynolds numbers. The main result is the Marangoni forces as a function of the prescribed velocity $\bar{U}$. The swimming condition (18) or (25) gives an additional force balance relation between Marangoni forces and $\bar{U}$, which has to be satisfied in the swimming state and determines the swimming speed $\bar{U}=\bar{U}_\mathrm{swim}$ as a function of Peclet number $\mathrm{Pe}$ and Biot number $\bar{k}$. In general, the swimming velocity increases with $\mathrm{Pe}$ and decreases with $\bar{k}$.

First, we consider small $\mathrm{Pe}$, i.e., small surfactant emission rates and see whether a swimming state with spontaneously broken symmetry can exist. For $\bar{k}\approx 0$, as appropriate for the PEG–alginate swimmer, we find from Eq. (30) that a solution for the swimming condition exists above a critical Peclet number $\mathrm{Pe}> \mathrm{Pe}_c \sim 8/\ln \bar{R} \rightarrow 0$, which approaches zero for large system sizes. Therefore, the symmetry is essentially always spontaneously broken in a large swimming vessel. Spontaneous symmetry breaking resulting in propulsion is possible by establishing an asymmetric surfactant concentration profile that is maintained by advection and can produce the necessary Marangoni forces. Equation (30) is valid only in the decoupled limit $\mathrm{Pe}\ll \bar{U}$. At the swimming bifurcation, we have $\mathrm{Pe} =\mathrm{Pe}_c \gg \bar{U}\approx 0$, however, such that the feedback of Marangoni flows onto the diffusion–advection problem has to be taken into account, and the decoupling approximation cannot be used. Then, Eq. (40) describes the Marangoni forces in the linear response regime, which further reduces the critical Peclet number to $\mathrm{Pe}_c \sim 1/(\ln \bar{R})^{3}\rightarrow 0$. In the presence of relevant evaporation $\bar{k}\gg 1$, as appropriate for camphor, the total Marangoni force is depressed according to Eq. (42) resulting in an increased $\mathrm{Pe}_c \sim \bar{k}^3/(\ln \bar{R})^{3} \rightarrow 0$, which is, however, still approaching zero for large swimming vessel sizes $\bar{R}$. Immediate propulsion in all experiments is in accordance with a bifurcation with a small $\mathrm{Pe}_c$. Moreover, we observe an intermittent stopping of the swimming motion only in the very end (after $20\, \mathrm{min}$ or more) before the swimming motion stops completely (because the fuel has been consumed). This confirms a small critical value $\mathrm{Pe}_c$ below which $\mathrm{Pe}$ drops only for very small emission current densities $\alpha$. Small irregularities can already break the symmetry and give rise to an avoided bifurcation and select a fixed swimming axis with respect to the particle orientation, which is also observed in the experiments.

For $\mathrm{Pe}> \mathrm{Pe}_c$, a spontaneously symmetry-broken swimming state with $\bar{U}_\mathrm{swim}>0$ exists. Because the Marangoni force Eq. (30) remains approximately linear up to $\bar{U}\sim O(1)$, as can also be seen in Fig. 10, the swimming velocity rises steeply for $\mathrm{Pe} > rsim \mathrm{Pe}_c$ and quickly enters the asymptotics for the advection-dominated regime $\bar{U}_\mathrm{swim} \gg 1$. Here, we find the swimming relations

44 $$\begin{aligned} \bar{U}_\mathrm{swim}&\sim \mathrm{Pe}^{3/5}&\text{ for }~ \bar{k}\ll \mathrm{Pe}^{1/5}, \end{aligned}$$

45 $$\begin{aligned} \bar{U}_\mathrm{swim}&\sim \bar{k}^{-3/4} \mathrm{Pe}^{3/4}&\text{ for }~ \bar{k}\gg \mathrm{Pe}^{1/5}. \end{aligned}$$

**Fig. 14:**
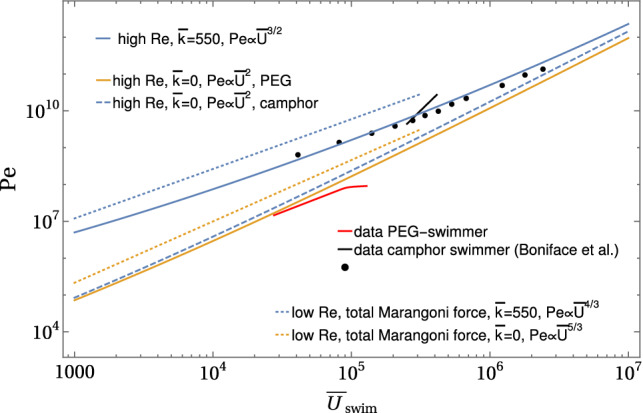
Different theory results for $\mathrm{Pe}$-$\bar{U}_\mathrm{swim}$ swimming relations in a double-logarithmic plot in comparison with experimental results on PEG–alginate swimmers (red lines, obtained from the data in Fig. 7) and camphor boats from Ref. [28] (black lines and dots; black line is time-dependent data, black dots are data at fixed times but for different radii *a*). PEG–alginate swimmers are described well by the high Reynolds number theory without evaporation (Biot number $\bar{k}=0$); camphor boats are best described by a high Reynolds number theory with Biot number $\bar{k}\approx 550$. The corresponding low Reynolds number theories give significantly lower swimming speeds

Also in this regime, we have $\mathrm{Pe} \gg \bar{U}_\mathrm{swim}$ such that Marangoni flows are strong, but this has little influence on the swimming speed because of the concentration boundary layer that forms in the advective regime. Evaporation is significant for $\bar{k} \gg \mathrm{Pe}^{1/5}$ and reduces the swimming speed because it reduces the driving Marangoni forces. The swimming relations (44) and (45) are shown in Fig. 14 as dotted yellow and dotted blue lines, respectively, together with the experimental results for our PEG–alginate swimmers and camphor boats from Boniface et al. [28]. We see clearly, that the experimentally observed swimming speed differs, because these swimmers operate at higher Reynolds numbers.

### High Reynolds numbers

We have developed a complete picture of the solution of problems (i)–(iii), i.e., diffusion–advection coupled to hydrodynamics for a prescribed swimmer velocity $\bar{U}$ at low Reynolds numbers, including evaporation. Low Reynolds numbers $\mathrm{Re} = 2\bar{U}/\mathrm{Sc} \ll 1$ are realized for $\bar{U} \ll \mathrm{Sc}/2$, which can still be much larger than unity for typical Schmidt numbers for surfactants in aqueous solutions (see Table 2). For the relevant Marangoni propulsion forces, the following picture has emerged from our analysis at low Reynolds numbers. There is a diffusive regime for $\bar{U}\ll 1$, which becomes modified by strong Marangoni flows for Peclet numbers $\mathrm{Pe}\gg \bar{U}$, and there is an advective regime for $\bar{U}\gg 1$, which is essentially unchanged in the presence of strong Marangoni flows for $\mathrm{Pe}\gg \bar{U}$ (see Fig. 12). Both regimes are modified in the presence of evaporation if the Biot number is $\bar{k} \ge 1$ in the diffusive regime and of $\bar{k} \gg \bar{U}^{1/3}$ in the advective regime (see Fig. 13).

High Reynolds numbers occur for large velocities $\bar{U} \gg \mathrm{Sc}/2$ and, therefore, always deep in the advective regime $\bar{U}\gg 1$. At low Reynolds numbers, the concentration boundary layer of dimensionless width $\varDelta \rho \sim \bar{U}^{-1/3}$ (see Eq. (31)) determines the results for the Marangoni forces in this advective limit [see Eqs. (27), (28), (29), (30), (40) and (43)].

In order to generalize to higher Reynolds numbers, we realize that the concentration boundary layer width is closely related to the Nusselt number. By definition (26), $\mathrm{Nu} = {-\partial _\rho \bar{c}_0(1)}/{\bar{c}_0(1)}$, the Nusselt number is an inverse extrapolation length, which we expect to be the inverse concentration boundary layer width,

46 $$\begin{aligned} \mathrm{Nu} \sim \frac{1}{\varDelta \rho }. \end{aligned}$$

The result $\mathrm{Nu} \sim \bar{U}^{1/3}$ from Eqs. (27) and (28) confirms this result both for constant flux and constant concentration boundary conditions at low Reynolds numbers, and we conjecture it to hold also at higher Reynolds numbers. Phenomenologically, the Nusselt number is well-studied also for high Reynolds number [61], both for heat ($\mathrm{Nu}_T$ in the following) and for mass transfer ($\mathrm{Nu}$ in the following, also Sherwood number $\mathrm{Sh}$ in the literature), and we can draw on these results in order to develop a theory for the concentration boundary layer and the Marangoni forces. Up to moderate Reynolds numbers $\mathrm{Re} \lesssim 200$, the physics is governed by additional laminar (viscous) boundary layers that appear around a sphere in fluid flow, which typically have a width $\bar{\delta } \propto \mathrm{Re}^{-1/2}$ [62, 63]. The viscous boundary layer scaling can be rationalized by generalizing our above scaling argument for the concentration boundary layer leading to Eq. (31). The important difference is that the velocity field close to the sphere changes from $v(\varDelta r) \sim U\varDelta r/a$ for Stokes flow to $v(\varDelta r) \sim U \varDelta r/\delta$ for laminar boundary layer flow with a no-slip boundary condition. This leads to

47 $$\begin{aligned} \varDelta \rho = \varDelta r/a \sim \bar{\delta }^{1/3} \bar{U}^{-1/3} \sim {\bar{U}}^{-1/2} \mathrm{Sc}^{1/6}. \end{aligned}$$

This scaling result is in accordance with phenomenological results for the Nusselt number by Ranz and Marshall [64] ($\mathrm{Re} = 2{\bar{U}}/\mathrm{Sc}$)

48 $$\begin{aligned} \mathrm{Nu}_T&= 1.0 + 0.3 \mathrm{Re}^{1/2} \mathrm{Pr}^{1/3} = 1.0 + 0.3 \sqrt{2}\bar{U}^{1/2} \mathrm{Sc}^{-1/2} \mathrm{Pr}^{1/3}, \nonumber \\ \mathrm{Nu}&= 1.0 + 0.3 \mathrm{Re}^{1/2} \mathrm{Sc}^{1/3} = 1.0 + 0.3 \sqrt{2}\bar{U}^{1/2} \mathrm{Sc}^{-1/6} \end{aligned}$$

($\mathrm{Re} = 2{\bar{U}}/\mathrm{Sc}$ and with $\mathrm{Sc}$ replacing the Prandtl number $\mathrm{Pr}$ for the mass transfer Nusselt number).

Because the concentration will drop in radial direction from its value at the surface *S* of the half-sphere to zero within the concentration boundary layer of width $\varDelta \rho$, and, thus, $1 = - \partial _\rho c(\rho =1) \sim c(\rho =1)/\varDelta \rho$ for constant flux boundary conditions, the scaling of the concentration boundary layer width (46) also gives rise to

49 $$\begin{aligned} \bar{c}(\rho \!=\!1,\theta ) \sim {\varDelta \rho } \sim \mathrm{Nu}^{-1}, \end{aligned}$$

i.e., the symmetry-breaking concentration level at the sphere is inversely proportional to the Nusselt number [generalizing Eq. (32)]. Therefore, the direct Marangoni force (23) should follow

50 $$\begin{aligned} \frac{\bar{F}_{\mathrm{M}}}{\mathrm{Pe}} \sim \int _0^\pi d\theta \cos \theta \bar{c}(1,\theta ) \sim {\mathrm{Nu}}^{-1} \sim \bar{U}^{-1/2} {\mathrm{Sc}}^{1/6} \end{aligned}$$

at higher Reynolds numbers. The total Marangoni force does no longer follow from a reciprocal theorem. In terms of an energy balance, the reciprocal theorem can be interpreted as the absence of mutual dissipation between swimming flow and Marangoni flow [58]. Therefore, the power input by surface Marangoni stresses into the swimming flow is, transmitted *without loss* as power input by the Marangoni flow force onto the swimmer. For higher Reynolds numbers, the mutual dissipation is no longer zero but there are additional viscous terms appearing, which are connected to the vorticity of the flow. This suggests that the Marangoni stresses at the interface become less effective in generating a Marangoni flow force because of this additional dissipation. Therefore, we simply neglect the Marangoni flow force (or assume that the Marangoni flow force is sub-dominant) and only consider the direct Marangoni force (50) at high Reynolds numbers, in the following.

Likewise, the existence of viscous boundary layers around the half-sphere modifies the drag force. On phenomenological grounds, it has been suggested that $F_\mathrm{D} = D_c \frac{\pi }{2}\mu a U$ with $D_c\simeq 6\mathrm{Nu}_T$ [65], where $\mathrm{Nu}_T$ is the Nusselt number for heat transport, resulting in

51 $$\begin{aligned} \bar{F}_\mathrm{D} = -3\pi \bar{U} \mathrm{Nu}_T. \end{aligned}$$

Using the Ranz and Marshall correlation (48), we find from the force balance $\bar{F}_\mathrm{D} +\bar{F}_\mathrm{M}=0$

52 $$\begin{aligned} \mathrm{Pe}&\approx 3 \mathrm{Nu}\mathrm{Nu}_T \bar{U}_\mathrm{swim}, \nonumber \\ \bar{U}_\mathrm{swim}&\sim \mathrm{Sc}^{1/3} \mathrm{Pr}^{-1/6} \mathrm{Pe}^{1/2} \end{aligned}$$

in the absence of evaporation. In the presence of evaporation, we use $\bar{F}_\mathrm{M} \sim \left. \bar{F}_\mathrm{M}\right| _{\bar{k}=0} {\mathrm{Nu}}/{(\bar{k}+\mathrm{Nu})}$ [cf. Eq. (43)] to find

53 $$\begin{aligned} \mathrm{Pe}&\approx 3 (\bar{k}+\mathrm{Nu})\mathrm{Nu}_T \bar{U}_\mathrm{swim}, \nonumber \\ \bar{U}_\mathrm{swim}&\sim \bar{k}^{-2/3} \mathrm{Sc}^{1/3} \mathrm{Pr}^{-1/2} \mathrm{Pe}^{2/3}. \end{aligned}$$

Both results (52) and (53) are also shown in Fig. 14 together with the experimental data on PEG–alginate and camphor Marangoni boats.

## Comparison with experiment

Force balance for low and high Reynolds numbers results in a characteristic $\mathrm{Pe}$-$\bar{U}_\mathrm{swim}$-relation for the swimmer with characteristic power laws. For low Reynolds numbers, these are relations (44), $\bar{U}_\mathrm{swim} \propto \mathrm{Pe}^{3/5}$, without evaporation and (45), $\bar{U}_\mathrm{swim} \propto \mathrm{Pe}^{3/4}$, in the presence of strong evaporation. For higher Reynolds numbers, we find relations (52), $\bar{U}_\mathrm{swim} \propto \mathrm{Pe}^{1/2}$ without evaporation and (53), $\bar{U}_\mathrm{swim} \propto \mathrm{Pe}^{2/3}$ for strong evaporation. Also, the experiment on PEG–alginate swimmers and the camphor boats from Boniface et al. [28] take place at higher Reynolds numbers; parameter value estimates for these experiments are summarized in Table 3, the resulting dimensionless parameters in Table 2. Our experimental results for the mass release mass $\dot{m}(t)$ as a function of time (see Fig. 7(middle)) and the corresponding swimming velocity $U_\mathrm{swim}(t)$ (see Fig. 7(right)) of the PEG–alginate swimmers give the red line in Fig. 14 in the $\mathrm{Pe}$-$\bar{U}_\mathrm{swim}$ parameter plane. We also show experimental results for camphor boats from Boniface et al. [28] (black data points from experiments varying the radius and black line from time-dependent swimming data). Figure 14 compares these experimental results with our theoretical results for the appropriate parameter values for the PEG–alginate swimmers and the camphor boats from Tables 2 and 3.

**Table 3:** Estimates of experimental parameters

Parameter	PEG–alginate swimmer	Camphor boat[28]
Radius *a*	$1500 \,{\upmu \mathrm{m}}$	$1000-15000\, {\upmu \mathrm{m}}$
Diffusion constant *D*	$350 \,{\upmu \mathrm{m}^2/s}$	$720 \,{\upmu \mathrm{m}^2/s}$
Surface tension reduction $\kappa = - {\varDelta \gamma }/{\varDelta c}$	$2.7\times 10^{-4} \,{\mathrm{m}^3/s^2}$	$2\times 10^{-2} \,{\mathrm{m}^3/s^2}$
Mass loss per time $\dot{m}= c\pi a^2\alpha$	$c_\mathrm{half-sph}=2$	$c_\mathrm{disk}=1$
	$0.01-0.25 \times 10^{-6} \,\mathrm{g/s}$	$(0.76 \times 10^{-6} \, \mathrm{g/s} )\left( {a}/{4\mathrm{mm}}\right) ^2$

The swimming relations in Fig. 14 are the main result of the paper. For the PEG–alginate swimmer (red line) we see good agreement between the high Reynolds number theory in the absence of evaporation, i.e., with Biot number of $\bar{k} =0$ (yellow line). The corresponding low Reynolds number theory (dotted yellow line) gives significantly lower swimming velocities at the same Peclet number. This outcome is what we expected based on the above estimate of moderate Reynolds numbers $\mathrm{Re} \sim 60$ for the PEG–alginate swimmers and based on the non-volatility of PEG. We also see that the slower second phase of the swimming motion of the PEG–alginate swimmers is described slightly better by our theory (left part of the red line in Fig. 14), which is in accordance with our initial observation that the time constants for mass release and swimming velocity agree only in the second phase. In the first phase, the Peclet number $\text {Pe}$ necessary to achieve the measured swimming velocity is slightly *lower* than predicted by our theory, i.e., a more efficient propulsion. This is a hint that some of our theoretical assumptions could be violated during the first phase, for example, regarding the adsorption equilibrium, which might not yet be established starting from an initially “empty” air–water interface, which could give rise to steeper concentration gradients and more efficient propulsion.

For volatile camphor, a Biot number of $\bar{k} \approx 550$ has been suggested in Ref. [26], which we use in Fig. 14 to compare with the experimental data of Boniface et al. on camphor disks [28] (black data points and black line). Again, we obtain good agreement with the high Reynolds number theory (blue line). The disk geometry differs from the half-spherical geometry we discussed in detail, but we expect that the swimming relation will only differ by numerical factors of order unity. The corresponding low Reynolds number theory (dotted blue line) significantly underestimates swimming velocities, and a theory without evaporation (dashed blue line) overestimates swimming velocities.

## Discussion and conclusion

We presented an experimental realization of alginate capsule self-propulsion at the air–water interface by loading the alginate capsule with surfactant molecules during synthesis. Self-propulsion of these capsule swimmers is based on a Marangoni boat mechanism. Alginate is bio-compatible and widely used for capsule production, which are interesting aspects for further applications. The versatile and simple synthesis strategy allowed us to identify various substances that can propel alginate capsules, see Table 1. PEG surfactants exhibit the best propulsion properties: for PEG-300, we find a fast and sustained motion with swimming speeds $U_\mathrm{swim} \sim 2-3 \,\mathrm{cm/s}$ over $20\, \mathrm{min}$ and more. The swimming speed corresponds to several swimmer diameters per second and is comparable or superior to other self-phoretic or microswimmers [4] or active liquid droplets [16]. In general, we find prolonged propulsion only if spreading molecules are water-soluble as the PEG molecules are; then the air–water interface can regenerate by the fuel being dissolved in water. Evaporation from the air–water interface is another mechanism to achieve regeneration, which is utilized in camphor boats [26–28, 32]. We conclude that a mechanism that regenerates the air–water interface, such as water-solubility or evaporation of surfactants, is crucial for prolonged propulsion.

We could produce alginate swimmers down to radii of several hundreds of micrometers, which is slightly above the realm of low Reynolds numbers. The future work could address further miniaturization of capsules.

Starting from low Reynolds numbers, we developed a theory for Marangoni boat propulsion of a completely symmetric, half-spherical, surfactant emitting swimmer. The theoretical description comprises the coupled problems of surface tension reduction by surfactant adsorption at the air–water interface including the possibility of surfactant evaporation, fluid flow (both Marangoni flow and flow induced by swimmer motion), diffusion and advection of the surfactant. In particular, advection is systematically included in our approach and turns out to be essential for all swimmer velocities $U \gg D/a$ ($\bar{U} \gg 1$). These three problems are first solved for prescribed swimmer velocity *U*; the actual swimming velocity $U_\mathrm{swim}$ is determined by force balance between the drag force, the direct Marangoni force from the surface tension contribution at the air–water–swimmer contact line, and the Marangoni flow force. We find that Marangoni flows can either act to increase the direct Marangoni force (at low velocities) or to increase the drag (at higher velocities). For low Reynolds numbers, all theoretical results are supported by numerical FEM simulations. Non-dimensionalization shows that the swimmer is controlled by two dimensionless control parameters, the Peclet number (21), which is the dimensionless emission rate of surfactant, and the Biot number (22), which is the dimensionless evaporation rate. Evaporation is practically absent for PEG, but strong for other frequently studied Marangoni boat swimmers, such as camphor boats [26].

We showed that a spontaneous symmetry breaking, i.e., a spontaneous transition into a swimming state is possible also for a completely symmetric swimmer above a critical Peclet number. Spontaneous symmetry breaking resulting in propulsion is possible by establishing an asymmetric surfactant concentration profile that is maintained by advection. We find that the critical Peclet number for this transition approaches zero logarithmically for large system sizes, $\mathrm{Pe}_c \propto 1/(\ln \bar{R})^{3}$. The possibility of such a spontaneous symmetry breaking has been pointed out for autophoretic swimmers [9, 18] and liquid Marangoni swimmers [17] before. Also in these systems, advection by the surrounding fluid can maintain the necessary concentration gradients in fields and/or concentrations.

In Eqs. (44) and (45), we obtain the power laws governing the swimming velocity as a function of Peclet and Biot number, which are $\bar{U}_\mathrm{swim} \propto \mathrm{Pe}^{3/5}$, without evaporation (PEG) and $\bar{U}_\mathrm{swim} \propto \bar{k}^{-3/4} \mathrm{Pe}^{3/4}$, in the presence of strong evaporation (camphor). This demonstrates that additional evaporation reduces swimming speed.

Experimentally realizable PEG–alginate or camphor swimmers are operating at moderate Reynolds numbers around 60 or more. Accordingly, we generalized the theoretical approach to higher Reynolds numbers by using the concept of the Nusselt number, for which many results at higher Reynolds numbers are known phenomenologically. This might also account for some effects related to the formation of vortices around the swimmer during propulsion at higher Reynolds numbers (see PIV-results in Fig. 6 and Ref. [29]). Finally, we obtained the swimming relations (52) and (53), which give $\bar{U}_\mathrm{swim} \propto \mathrm{Pe}^{3/4}$, without evaporation (PEG) and $\bar{U}_\mathrm{swim} \propto \bar{k}^{-2/3} \mathrm{Pe}^{1/2}$, in the presence of strong evaporation (camphor). We find a good quantitative fit (without any free fitting parameters) with our own experimental results on PEG–alginate swimmers and the results of Ref. [28] on camphor swimmers in Fig. 14. This is the main result of this paper. The future work should extend the numerical approach to higher Reynolds numbers in order to verify our scaling results for the swimming relation using, for example, the methods introduced in Ref. [37]. There are several aspects of the self-propulsion of PEG–alginate capsules, where we presented first experimental results but which deserve a much more detailed investigation in future work: curved trajectories, interactions with container walls, and swimmer-swimmer interaction.

Curved trajectories as observed in Figs. 4 and 5 with a swimming direction of the swimmer that is, at least, weakly linked to its orientation, while the orientation of the swimmer is slowly turning can only be explained by small asymmetries of capsules induced by irregularities in the pore structure. This view is supported by the individual character of the turning characteristics of different swimmers (see Fig. 5). Future work should explore the relation between capsule irregularities and turning statistics in more detail. Experimentally, purely rotary systems could be constructed [42].

PEG–alginate swimmers are repelled by walls. In normal collisions we observe direction reversal without reorientation of the swimmer. In the framework of the Marangoni boat mechanism, this can be explained by an accumulation of surfactant emitted by the swimmer in front of the wall because of the zero flux boundary condition at the wall. Surfactant accumulation creates a gradient in surfactant concentration toward the wall, and the swimmer reverses direction if advection and accumulation balance without changing its orientation. This behavior is similar to what has been observed for asymmetric [24] and symmetric [25] camphor boats [22]. During the collision the orientation of the swimmer particle does not change, while the swimming direction reverses; therefore, the swimming direction also reverses relatively to the particle orientation. This is consistent with a weak symmetry breaking by small irregularities in the pore distribution, which give rise to many possible metastable propulsion directions. A perturbation as during surfactant accumulation and direction reversal at the wall can easily cause a change between these propulsion directions. There are more oblique collisions (see Fig. 5), which take longer and can feature a reorientation of the swimmer. The underlying mechanisms could be similar to the reorientation mechanisms of self-diffusiophoretic swimmers [13, 14] but this issue also requires future work.

Finally, we have experimental evidence that PEG–alginate swimmers interact with each other via their surfactant concentration fields. Similar observations have been made already in Refs. [22, 40, 66], mostly in channel geometries. We monitored different collisions between swimmers, where we could find both attraction and repulsion. In the framework of the Marangoni boat mechanisms, where swimmers prefer to move opposite to surfactant concentration gradients, we expect that swimmers are repelled by their surfactant tails, which represent traces of high concentration. This predicts a kind of “chemo-repellent” behavior with respect to the tails. For the interaction between swimmers, the concentration dependence of the surface activity $\kappa$ [the $c_0$-dependence in Eq. (5)] can also play an important role [40]. The topic of swimmer interactions appears to be very rich and important for applications regarding the swarming of PEG–alginate swimmers; it deserves a much more detailed investigation in future work.

## Numerical methods

For $\mathrm{Pe} \ll \bar{U}$, the Marangoni flow problem decouples and the advection problem becomes axisymmetric. Then, $c=c(\rho ,\theta )$ only depends on the radial coordinate and one angular coordinate and we solve the diffusion–advection problem on a two-dimensional rectangular domain in the $\rho$-$\cos \theta$ plane. Typically we use $\rho < 30$ and FEM-routines from Wolfram MATHEMATICA; we use irregular triangular meshes with a mean area of mesh elements of 0.00015 in the $\rho$-$\cos \theta$ plane (the maximal area is 0.005). We checked that discretization effects are negligible.

For $\mathrm{Pe} \gg \bar{U}$, we have to solve the coupled problems of Marangoni flow field (iib) and diffusion–advection equation (iii) with the appropriate boundary conditions from the adsorption problem (i). Numerically, we only address low Reynolds numbers for the fluid flow such that the Stokes equation applies. For the coupled problem we use an iterative scheme of three-dimensional FEM solutions both for Marangoni flow and for diffusion–advection, again employing FEM-routines from Wolfram MATHEMATICA. For the Stokes flow, we use the analytical result [Eqs. (9a) and (9b)]. In the iterative scheme we solve for the Marangoni flow field (iib) starting from an initial guess for the concentration profile (typically $\bar{c}^{(0)}(\rho ) =1/\rho$). Then, we use this Marangoni flow field in the advecting fluid flow field in the FEM solution $\bar{\varvec{v}}_\mathrm{M}^{(1)}(\varvec{\rho })$ of the diffusion–advection equation (iii), which gives in turn an improved approximation $\bar{c}^{(1)}(\varvec{\rho })$ for the concentration profile. With this improved approximation we go back into solving for the Marangoni flow field (iib) to obtain the next iteration $\bar{\varvec{v}}_\mathrm{M}^{(2)}(\varvec{\rho })$, and so on. This results in improving approximations $\bar{c}^{(n)}(\varvec{\rho })$ and $\bar{\varvec{v}}_\mathrm{M}^{(n)}(\varvec{\rho })$, and the iteration typically converges within $n=5-10$ iterations to the final Marangoni flow field and surfactant concentration field. The iterative approach has the advantage that the Marangoni boundary condition in the fluid flow problem (iib) is a fixed one at each iterative step and only adjusts over the iteration; the coupling of the two problems is correctly established over the iteration. Similar iterative numerical schemes for coupled problems have been applied successfully in Refs. [67, 68]. The iterative scheme works well up to $\mathrm{Pe}\sim 50$ for the system sizes we use. As outlined in the section on strong Marangoni flow in the limit $\mathrm{Pe} \gg \bar{U}$, Marangoni flows are fully established on the scale $\rho _\mathrm{M} \sim \mathrm{Pe}$, which is growing with the Peclet number. Therefore, numerical problems typically arise for higher Peclet numbers when the Marangoni roll becomes strongly distorted by the system boundaries, which gives rise to numerical instabilities and a failure of the iteration procedure. This is why the data presented in Fig. 12 is limited to the range $\mathrm{Pe} = 0-50$.

The FEM solution of the stationary equations (iib) and (iii) is obtained on a cylindrical or cubical irregular tetrahedral mesh. We use cubical volumes (for example, with edge length 14 in $\bar{x}\bar{z}$-plane and height 7 in $\bar{y}$-direction in Fig. 12) for the FEM calculations. Cylindrical volumes can also be easily implemented. The maximal volume of mesh elements is 0.2, the mean volume is 0.01. Mesh volumes are smaller ($<0.005$) in the region $-1<\bar{y}<0$ below the interface to capture Marangoni advection. Again, we checked that discretization effects are small. Because of the mirror symmetry $\bar{x}\rightarrow -\bar{x}$, we only need to solve on half-cubes $\bar{x}>0$ and apply Neumann boundary conditions $\left. \partial _{\bar{x}} \bar{c}\right| _{\bar{x}=0}=0$ and $\left. \partial _{\bar{x}} \bar{\varvec{v}}_\mathrm{M}\right| _{\bar{x}=0}=0$ to enforce the mirror symmetry. The boundary conditions at the outer boundaries are Dirichlet conditions for the concentration $\bar{c}=0$ and the Marangoni flow $\bar{\varvec{v}}_\mathrm{M}=0$. For sufficiently large cubes or cylinders these boundary conditions should not matter but we still have finite size effects, in particular at larger Peclet numbers $\mathrm{Pe}>50$ as explained above. In particular, our systems are always several particle radii long in every direction such that strong confinement effects as observed in Ref. [37] for system sizes below two particle radii should be absent.

We are interested in the resulting symmetry-breaking Marangoni forces caused by a symmetry-breaking swimming motion as a function of the velocity $\bar{U}$. At small $\bar{U}$, there is the problem that artificial symmetry breaking from lattice irregularities/defects is often larger than symmetry breaking by swimming. Therefore, we average all measured quantities over two simulations with $\bar{U}$ and $-\bar{U}$ to cancel artificial symmetry-breaking effects. This step is crucial to obtain accurate results.

## Footnotes

1 In many publications on the diffusion–advection problem, such as Refs. [54–56] but also in Refs. [10, 28, 37–39], $\bar{U}$ is called the Peclet number. Here, we define the Peclet number as $\mathrm{Pe} \equiv \bar{U}_{\alpha }$, i.e., by the characteristic velocity $\bar{U}_{\alpha } = {\kappa \alpha a}/{D \mu }$, where a typical direct Marangoni force $F_\mathrm{M} \sim \kappa a^2 \partial _rc(r=a)\sim \kappa a^2\alpha /D$ is balanced by the typical Stokes drag force $F_D \sim \mu a U$. The Peclet number is a dimensionless measure of propulsion strength with this definition.

## References

[1] Purcell EM (1977). Am. J. Phys..

[2] Lauga E, Powers TR (2009). Rep. Prog. Phys..

[3] Anderson J (1989). Ann. Rev. Fluid Mech..

[4] Ebbens SJ, Howse JR (2010). Soft Matter.

[5] Illien P, Golestanian R, Sen A (2017). Chem. Soc. Rev..

[6] Popescu MN, Dietrich S, Oshanin G (2009). J. Chem. Phys..

[7] M.N. Popescu, M. Tasinkevych, S. Dietrich, EPL **95**, 28004 (2011)

[8] Mozaffari A, Sharifi-Mood N, Koplik J, Maldarelli C (2016). Phys. Fluids.

[9] Michelin S, Lauga E (2014). J. Fluid Mech..

[10] Yariv E, Michelin S (2015). J. Fluid Mech..

[11] Sabass B, Seifert U (2010). Phys. Rev. Lett..

[12] Baraban L, Tasinkevych M, Popescu MN, Sanchez S, Dietrich S, Schmidt OG (2012). Soft Matter.

[13] Uspal WE, Popescu MN, Dietrich S, Tasinkevych M (2015). Soft Matter.

[14] Bayati P, Popescu MN, Uspal WE, Dietrich S, Najafi A (2019). Soft Matter.

[15] Scriven LE, Sternling CV (1960). Nature.

[16] Herminghaus S, Maass CC, Krüger C, Thutupalli S, Goehring L, Bahr C (2014). Soft Matter.

[17] Izri Z, Van Der Linden MN, Michelin S, Dauchot O (2014). Phys. Rev. Lett..

[18] Michelin S, Lauga E, Bartolo D (2013). Phys. Fluids.

[19] Yoshinaga N, Nagai KH, Sumino Y, Kitahata H (2012). Phys. Rev. E.

[20] Schmitt M, Stark H (2016). Phys. Fluids.

[21] Tomlinson C (1864). London, Edinburgh Dublin Philos. Mag. J. Sci..

[22] Nakata S, Nagayama M, Kitahata H, Suematsu NJ, Hasegawa T (2015). Phys. Chem. Chem. Phys..

[23] Renney C, Brewer A, Mooibroek TJ (2013). J. Chem. Educ..

[24] Hayashima Y, Nagayama M, Nakata S (2001). J. Phys. Chem. B.

[25] Nagayama M, Nakata S, Doi Y, Hayashima Y (2004). Physica D.

[26] Soh S, Bishop KJ, Grzybowski BA (2008). J. Phys. Chem. B.

[27] V.S. Akella, D.K. Singh, S. Mandre, M.M. Bandi, Phys. Lett. A **382**, 1176 (2018), arXiv:1701.06775v1

[28] Boniface D, Cottin-Bizonne C, Kervil R, Ybert C, Detcheverry F (2019). Phys. Rev. E.

[29] S. Sur, H. Masoud, J.P. Rothstein, Phys. Fluids **31**, 102101 (2019)

[30] Löffler RJG, Hanczyc MM, Gorecki J (2019). Phys. Chem. Chem. Phys..

[31] Wang L, Yuan B, Lu J, Tan S, Liu F, Yu L, He Z, Liu J (2016). Adv. Mater..

[32] Suematsu NJ, Sasaki T, Nakata S, Kitahata H (2014). Langmuir.

[33] Bush JW, Hu DL (2005). Annu. Rev. Fluid Mech..

[34] Dietrich K, Jaensson N, Buttinoni I, Volpe G, Isa L (2020). Phys. Rev. Lett..

[35] Würger A (2014). J. Fluid Mech..

[36] Gidituri H, Panchagnula MV, Pototsky A (2019). Soft Matter.

[37] Jafari Kang S, Sur S, Rothstein JP, Masoud H (2020). Phys. Rev. Fluids.

[38] Lauga E, Davis AMJ (2012). J. Fluid Mech..

[39] Vandadi V, Kang SJ, Masoud H (2017). J. Fluid Mech..

[40] Heisler E, Suematsu NJ, Awazu A, Nishimori H (2012). Phys. Rev. E.

[41] Iida K, Kitahata H, Nagayama M (2014). Physica D.

[42] Koyano Y, Gryciuk M, Skrobanska P, Malecki M, Sumino Y, Kitahata H, Gorecki J (2017). Phys. Rev. E.

[43] H. Thiele, *Histolyse und Histogenese: Gewebe und ionotrope Gele; Prinzip einer Strukturbildung* (Akademische Verlagsgesellschaft, 1967)

[44] Leick S, Kemper A, Rehage H (2011). Soft Matter.

[45] Klein J, Stock J, Vorlop KD (1983). Eur. J. Appl. Microbiol. Biotechnol..

[46] L. Cao, W. Lu, A. Mata, K. Nishinari, Y. Fang, Carbohydr. Polym. **242**, 116389 (2020) 10.1016/j.carbpol.2020.116389 32564839

[47] Nakata S, Iguchi Y, Ose S, Kuboyama M, Ishii T, Yoshikawa K (1997). Langmuir.

[48] Johnson TA, Patel VC (1999). J. Fluid Mech..

[49] Higuchi T (1961). J. Pharm. Sci..

[50] Higuchi T (1963). J. Pharm. Sci..

[51] M.J. Rosen, J.T. Kunjappu, *Reduction of Surface and Interfacial Tension by Surfactants* (John Wiley & Sons, Ltd, 2012), chap. 5, pp. 235–271, ISBN 9781118228920

[52] Li B, Geeraerts G, Joos P (1994). Colloids Surfaces A Physicochem. Eng. Asp..

[53] Masoud H, Stone HA (2014). J. Fluid Mech..

[54] Acrivos A (1960). Phys. Fluids.

[55] Acrivos A, Taylor TD (1962). Phys. Fluids.

[56] Acrivos A, Goddard JD (1965). J. Fluid Mech..

[57] L. Leal, *Laminar Flow and Convective Transport Processes: Scaling Principles and Asymptotic Analysis* (Butterworth-Heinemann, 1992). 9780750691178

[58] H. Ender, J. Kierfeld, Eur. Phys. J. E **44**, 4 (2021) 10.1140/epje/s10189-021-00034-9 PMC7880915 33580288

[59] Le Roux S, Roché M, Cantat I, Saint-Jalmes A (2016). Phys. Rev. E.

[60] Roché M, Li Z, Griffiths IM, Le Roux S, Cantat I, Saint-Jalmes A, Stone HA (2014). Phys. Rev. Lett..

[61] Michaelides EE (2003). J. Fluids Eng..

[62] F. White, *Viscous Fluid Flow*, McGraw-Hill International edition. (McGraw-Hill, 2006). 9780071244930

[63] Schlichting H, Gersten K (2016). Boundary-Layer Theory.

[64] W.E. Ranz, W.R. Marshall, *Evaporation from drops* (1952)

[65] Duan Z, He B, Duan Y (2015). Sci. Rep..

[66] Kohira MI, Hayashima Y, Nagayama M, Nakata S (2001). Langmuir.

[67] Boltz HH, Kierfeld J (2015). Phys. Rev. E.

[68] Wischnewski C, Kierfeld J (2018). Phys. Rev. Fluids.

